# Enhanced antibiotic distribution strategies and the potential impact of facial cleanliness and environmental improvements for the sustained control of trachoma: a modelling study

**DOI:** 10.1186/s12916-016-0614-6

**Published:** 2016-05-19

**Authors:** Amy Pinsent, Matthew J. Burton, Manoj Gambhir

**Affiliations:** Department of Epidemiology and Preventive Medicine, Monash University, Melbourne, Australia; International Centre for Eye Health, Department of Clinical Research, London School of Hygiene & Tropical Medicine, London, UK

**Keywords:** Mathematical modelling, Trachoma, Mass drug administration, GET 2020, Control, Elimination

## Abstract

**Background:**

Despite some success in controlling trachoma with repeated mass drug administration (MDA), some hyperendemic regions are not responding as fast as anticipated. Available data suggests that individuals with higher bacterial infection loads are less likely to resolve infection following a single dose of treatment, and thus remain a source of re-emergent infection following treatment. We assessed the potential impact of a new double-dose antibiotic distribution strategy in addition to enhanced facial cleanliness (F) and environmental improvements (E).

**Methods:**

Using a within-community mathematical model of trachoma transmission we assessed the impact of a new double-dose antibiotic distribution strategy given 2 weeks apart, with and without enhanced F&E. We compared the annual double-dose strategy to single-dose annual MDA treatment in hyper-, meso- and hypoendemic settings, and to biannual MDA at 6-monthly intervals in hyperendemic communities.

**Results:**

The findings from our mathematical model suggest that implementing the new double-dose strategy for 5 years or less was predicted to control infection more successfully than annual or 6-monthly treatment. Infection was controlled more readily if treatment was combined with enhanced F&E. The results appeared robust to variation in a number of key epidemiological parameters. To have long-term impact on transmission, enhanced F&E is essential for high transmission settings.

**Conclusion:**

Our current findings are based on simualtion modelling only, due to lack of epidemilogical data, however they do suggest that the  annual double-dose treatment strategy is encouraging for trachoma control. In high transmission settings, both MDA and enhanced F&E are needed for sustained control.

**Electronic supplementary material:**

The online version of this article (doi:10.1186/s12916-016-0614-6) contains supplementary material, which is available to authorized users.

## Background

Trachoma remains the most common infectious cause of blindness worldwide. It is estimated that 84 million people, mostly young children, have active disease, and 1.2 million people are blind from trachoma [1]. Blindness occurs as a result of repeated infection of the ocular surface with the bacterium *Chlamydia trachomatis*. The infection results in chronic, recurrent conjunctival inflammation (active trachoma), which then progresses to scarring [2]. This scarring leads to in-turning of the eyelashes, known as trachomatous trichiasis (TT), which traumatises the eye surface leading to corneal opacification and blindness [2].

To prevent sight loss from trachoma, effective long-term control of *C. trachomatis* infection and transmission is required [2]. The World Health Organization (WHO) aims to eliminate trachoma as a public health problem by 2020. To help achieve this, the Alliance for the Global Elimination of Trachoma by the year 2020 (GET 2020) was formed. The Alliance has two major elimination targets, the first of which is to reduce the prevalence of trachomatous inflammation, follicular (TF) in children aged 1–9 years old to < 5 % in all endemic regions by 2020. To help reach this goal, the WHO recommends the use of the SAFE strategy: surgery for trichiasis, antibiotics to treat infection, facial cleanliness and environmental improvements to suppress transmission [2]. Programmatic success is evaluated through follow-up surveys assessing prevalence of TF in 1–9-year-olds, following several annual antibiotic treatment rounds.

The current antibiotic of choice is oral azithromycin, which is distributed to entire endemic districts meeting the intervention criteria. The WHO recommends that treatment is repeated annually for an initial 3–5-year period, depending on the baseline disease prevalence [3]. Unfortunately, particularly in areas with a high baseline prevalence, the response to treatment at the community level has been relatively disappointing. In some areas which have been studied in detail, such as Kongwa, Tanzania [4], and Gurage, Ethiopia [5], more than seven rounds of treatment have been deployed; however, prevalence of active disease has not dropped below the target level of < 5 % in children aged 1–9 years to eliminate trachoma as a public health problem. This is supported by findings from a recent study, in which the authors suggested that countries should prepare for extended antibiotic timelines [6]. As the target date to eliminate trachoma as a public health problem by 2020 fast approaches, there is a pressing need to identify alternative, more effective antibiotic distribution strategies for highly endemic regions.

Individuals with the highest ocular chlamydial loads are most commonly young children, under 10 years of age [7]. Available data suggest that individuals with higher bacterial infection loads are less likely to resolve infection upon treatment than children with a lower starting load. Therefore, children with the highest bacterial loads pre-treatment can remain a source for re-emergent infection in the community [8]. Additionally, re-emergence of disease clusters have been reported around households of children identified as having high bacterial loads [9, 10]. Furthermore, simply increasing the dose of azithromycin for those with severe active trachoma (as a surrogate marker for higher infection loads) was not found to be more effective than a standard dose in resolving infection [9]. Similarly, treatment of individuals with severe active trachoma with two standard antibiotic doses given on consecutive days was also not found to be more effective than the standard single dose [11]. However, it is possible that the second dose applied in this trial on the second day did not have additional benefit, as it was administered when an individual probably still had a high bacterial load [11].

Therefore, in this study we suggest the implementation of an alternative double-dose antibiotic distribution strategy, where two doses of antibiotic are administered 2 weeks apart from one another. The rationale for this approach is that the probability of resolving infection following treatment is related to the load. The available data suggest that while some high-load infections do fully resolve following a single dose of treatment, a proportion of these high-load infections do not; however, they do transition into a lower-load category [8]. Here, the first dose of antibiotic treatment acts to reduce an individual’s bacterial load. Individuals are then re-treated 2 weeks after the first dose. Those that are still infected after the first treatment are likely to be in a lower-load state, and as such, they have a higher probability of fully resolving their infection following the second antibiotic dose.

Treatment of infection with antibiotics represents only one arm of the SAFE strategy. Indeed, a recent analysis suggested that particularly for highly endemic regions, repeated annual single-dose mass drug administration (MDA) alone is insufficient to eliminate infection, without lasting environmental improvements [6]. The quantitative impact of non-pharmaceutical interventions (NPIs) on trachoma transmission remains unclear. One recent study attempted to quantify the impact of facial cleanliness (F) and environmental improvements (E) in helping to reduce transmission and the odds of acquiring infection [12]. The authors suggested that the impact of improved sanitation and hygiene in helping to reduce trachoma transmission is currently underestimated and that it can help to reduce the odds of acquiring infection [12]. However, the mechanisms of transmission remain poorly defined, and there are few randomised control trials in this area to support or quantify the role of F&E on trachoma.

Here, we propose a development of the existing F&E interventions, which we refer to as ‘enhanced F&E’. In this trial scenario, enhanced F&E would be developed through intensive observational studies, which document human behaviour, its determinants and the hygiene practices adopted by individuals. This focused ethnographic approach will thus document in detail key behaviours and possible transmission routes relevant to trachoma transmission. Through this, targeted F&E interventions will be developed in partnership with the communities to generate promotional activities to prevent these pathways occurring. It is this specific targeting of interventions and promotional activity informed by ethnographic research that defines enhanced F&E.

Previous mathematical modelling studies have suggested that in areas of high transmission intensity at least two rounds of single-dose antibiotic treatment at 6-monthly intervals may be needed to help control infection [5, 13, 14]. However, one clinical trial in high prevalence communities reported that despite biannual treatment for 3 years infection re-emerged [5]. The logistics and cost of distributing treatment at 6-monthly intervals requires considerable resources. If the second dose is given in quick succession there are likely to be some savings in cost relative to giving MDA at 6-monthly intervals; for example, less overall preparation and management costs, it would only be necessary to sensitise and mobilise the community once a year, and there may also be some transport cost savings if the distribution team is still in the same area.

Here, we explore the potential community-level impact of the double-dose antibiotic distribution strategy administered on an annual basis to help reduce transmission of the pathogen and burden of disease within the community. We examine the projected impact of reductions in transmission that may occur as a result of the introduction of enhanced F&E interventions, as defined previously. We assess our findings across a range of key parameters, including: frequency of treatment, coverage of treatment, differential treatment efficacy and a range of reductions in the transmission rate.

## Methods

### Model structure

**Fig. 1:**
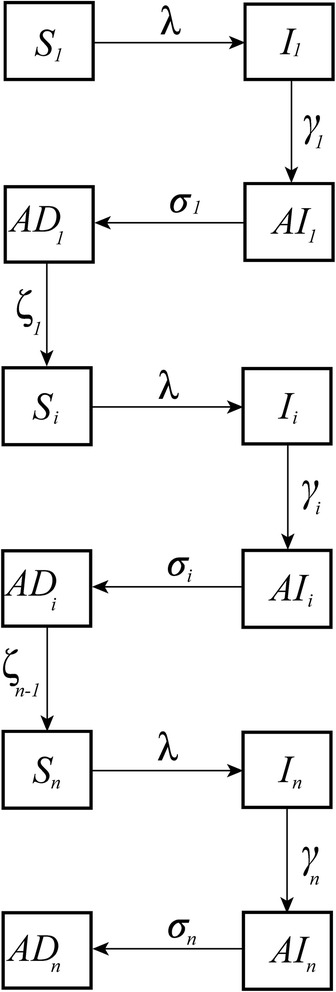
A flow diagram of the SIAIAD trachoma infection transmission model. Individuals begin as susceptible to their first infection (*S*), become infected at a rate *λ*, progress to the incubating class (*I*) and leave at a rate *γ*_i_, which does not depend on the previous number of infections they have had. They progress into the infected with active disease compartment (*AI*) and leave at a rate *σ*_i_, which does depend on the previous number of infections experienced. They lastly progress into the active disease compartment (*AD*), where they are not infectious but still have identifiable active disease, individuals recover from active disease and become susceptible to infection again at a rate *ζ*_i_, which depends on the number of infections experienced

The mathematical model developed represents ocular infection events with *C. trachomatis* within a community. The model is an extension of a susceptible, infected, susceptible (SIS) transmission model. We include two extensions to this framework: firstly, the addition of a short latent period; and secondly, a compartment that considers individuals who present with active disease but do not test PCR positive. This four-state structure is analogous to that used by Grassly et al. [15]. Immunity to re-infection with trachoma is short term; therefore, individuals can be repeatedly infected throughout their lifetime. To account for this we follow the ‘ladder of infection’ model structure initially presented by Gambhir et al. [16, 17]. A flow diagram of the infection process is presented in Fig. 1. We follow all individuals from their first infection onwards; however, after 100 infections, improved immunity with re-infection plateaus, and an individual’s first ten infections are considered high load (sensitivity of the results to these assumptions are presented in Additional file 1). In the absence of any empirical data we assumed that repeated infection did not alter an individual’s incubation period, or their susceptibility to re-infection. Further detail on the modelling methods is provided in Additional file 1.

We assumed a hyperendemic community had a PCR prevalence of infection > 20 %, a mesoendemic community had ≤ 20 % and a hypoendemic community had < 10 %. On a person-by-person basis, the relationship between the prevalence of infection and TF is not directly linear; however, at the population level this relationship is approximately linear.

### Modelling different intervention scenarios

We considered four different treatment scenarios. For hyperendemic communities, we modelled five annual rounds of MDA, with follow-up extending for 1 year after the final treatment. For the meso- and hypoendemic communities, we modelled three annual rounds of MDA for each setting, with follow-up extending for 3 years after the third treatment. A differential treatment efficacy for individuals with high and low bacterial loads was assumed for all intervention scenarios. Treatment efficacy was consistent across all rounds of treatment. Scenario one: single-dose annual MDA. Scenario two: the new alternative treatment regime of a double-dose of annual MDA, given in quick succession 2 weeks apart. Scenario three: single-dose annual MDA, with a once-off instantaneous drop in the transmission rate parameter *β* at the time of the first MDA treatment in year one. Since *β* is the product of the transmission probability and the contact rate between individuals, we consider this instantaneous drop in *β* to model the impact of the introduction of enhanced F&E within the community. This instantaneous drop in *β* occurred only once in the first year at the first round of treatment, but was sustained for the 6-year period. Further reductions in *β* after this first round of treatment were not modelled. Scenario four: double-dose annual MDA and a once-off instantaneous drop in *β* at time of first MDA treatment in year one (as described for scenario three). Again, here the instantaneous drop in *β* represents the introduction of enhanced F&E.

For the hyperendemic community, we also compared the new double-dose treatment strategy to biannual treatment at 6-monthly intervals.

### Sensitivity analysis

Given the limited availability of data to parameterise achievable reductions in the probability of transmission through enhanced F&E, we explored a range of reductions in *β*. We assessed a percentage reduction in *β* from 0 % to 50 %. This did not directly impact an individual’s bacterial load, but assumed that for any given bacterial load level the probability of infection transmitted to a susceptible individual in the community was lower following the implementation of enhanced F&E. For the baseline analysis, we assumed an instantaneous reduction in *β* at the first treatment time point. However, it is possible that even with a highly targeted F&E approach interventions will not be accessed by all members of the community equally, or at the same rate. We therefore also considered a non-instantaneous reduction in *β*. Instead, we assumed an exponential reduction in *β* throughout the intervention period, where, at each treatment time point, *β* was also reduced by a small amount. Thus, the cumulative reduction in *β* across the 3- or 5-year intervention period remained the same as when the instantaneous drop was considered, but at any given point in time prior to the last round of MDA the value of *β* considering a non-instantaneous drop would be higher than when the instantaneous drop was assumed.

Estimates of the efficacy of azithromycin are variable and can depend on the host’s bacterial load [8, 10]. Therefore, we explored a range of plausible treatment efficacy values. For those classified as having high bacterial loads (high loaders) efficacy of treatment was assumed to range between 40 % and 75 %. For those assumed to have low bacterial loads (low loaders) treatment efficacy was assumed to range between 70 % and 90 % (Additional file 1: Table S1). The level of treatment coverage achieved is likely to vary between settings, thus we assessed the impact of each intervention scenario under different coverage levels of 60–95 % (Additional file 1: Table S1). We also assessed how our findings vary according to the assumptions about when immunity to infection plateaus and the number of infections that are high-load infections (Additional file 1).

Lastly, our baseline model assumed that individuals in the active disease state were immune to re-infection. We performed a final sensitivity analysis to this assumption and assumed individuals in the active disease state had a 50 % probability of being re-infected; results are presented in Additional file 1.

## Results

### MDA alone

**Fig. 2:**
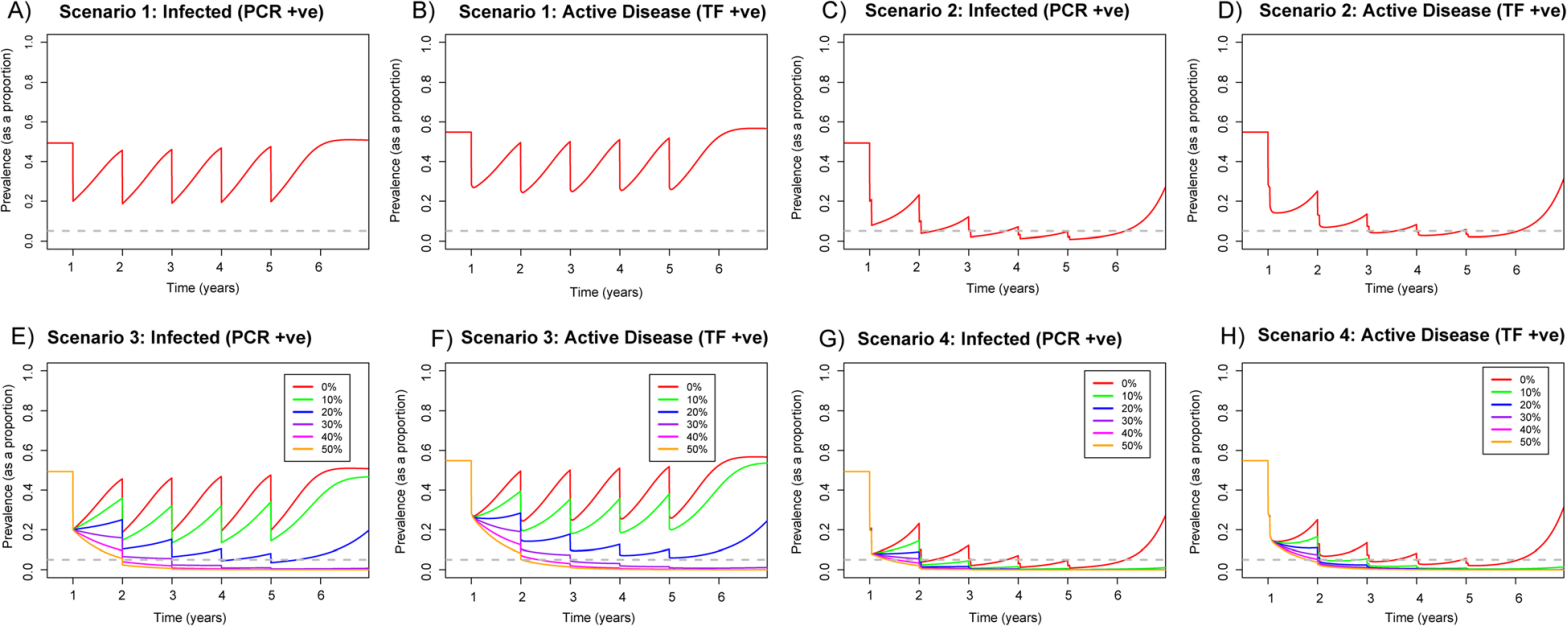
Prevalence of infection and active disease in 0–9-year-olds when comparing single-dose annual MDA to double-dose annual MDA (conducted 2 weeks apart) within a hyperendemic community for 5 years. We assumed 80 % coverage, and 65 % and 85 % efficacy of the antibiotic for high and low bacterial loaders, respectively. Scenario 1: **a** prevalence of infection and **b** active disease with single-dose annual MDA, with a 0 % decline in *β*. Scenario 2: **c** prevalence of infection and **d** active disease with double-dose annual MDA applied 2 weeks apart, with a 0 % decline in *β*. Scenario 3: **e** prevalence of infection and **f** active disease with single-dose annual MDA. Scenario 4: **g** prevalence of infection and **h** active disease with double-dose annual MDA applied 2 weeks apart. While also modelling an instantaneous drop in *β*, we present a range of reductions in *β. Different coloured lines* represent different percentage declines in the value of *β*, ranging from 0–50 %. *Grey dashed line* indicates 5 % prevalence, where < 5 % prevalence of active disease is the GET 2020 target level

For a hyperendemic community with intervention scenario two, infection and disease prevalence were reduced to less than 10 % following the first year of treatment (Fig. 2c, d), while with scenario one, prevalence was only reduced to 20 % and rebounded within 1 year. Across the five rounds we observed no long-term trends of an overall decline in prevalence within the hyperendemic community with a single annual dose (Fig. 2a, b), while with the double-dose treatment we saw year-on-year gains and the prevalence of disease was driven below 5 % following ten rounds of treatment. However, when treatment ceased in both scenarios infection re-emerged (Fig. 2a–d).

**Fig. 3:**
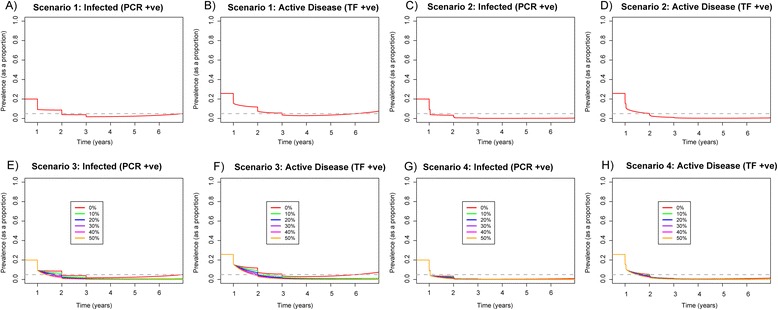
Prevalence of infection and active disease in 0–9-year olds when comparing single-dose annual MDA to double-dose annual MDA (conducted 2 weeks apart) within a mesoendemic community for 3 years. We assumed 80 % coverage, and 65 % and 85 % efficacy of the antibiotic for high and low bacterial loaders, respectively. Scenario 1: **a** prevalence of infection and **b** active disease with single-dose annual MDA, with a 0 % decline in *β*. Scenario 2: **c** prevalence of infection and **d** active disease with double-dose annual MDA applied 2 weeks apart, with a 0 % decline in *β*. Scenario 3: **e** prevalence of infection and **f** active disease with single-dose annual MDA. Scenario 4: **g** prevalence of infection and **h** active disease with double-dose annual MDA applied 2 weeks apart. While also modelling an instantaneous drop in *β*, we present a range of reductions in *β. Different coloured lines* represent different percentage declines in the value of *β*, ranging from 0–50 %. *Grey dashed line* indicates 5 % prevalence, where < 5 % prevalence of active disease is the GET 2020 target level

The MDA-induced prevalence decline in a mesoendemic community (Fig. 3a–d) was much larger than the hyperendemic setting. Within 1 year of treatment, prevalence of infection was reduced to 5 % in scenario two, while with scenario one it was reduced to 10 % (Fig. 3a, c). Following cessation of treatment re-emergence of infection was observed in scenario one (Fig. 3a, b), but not scenario two (Fig. 3c, d).

**Fig. 4:**
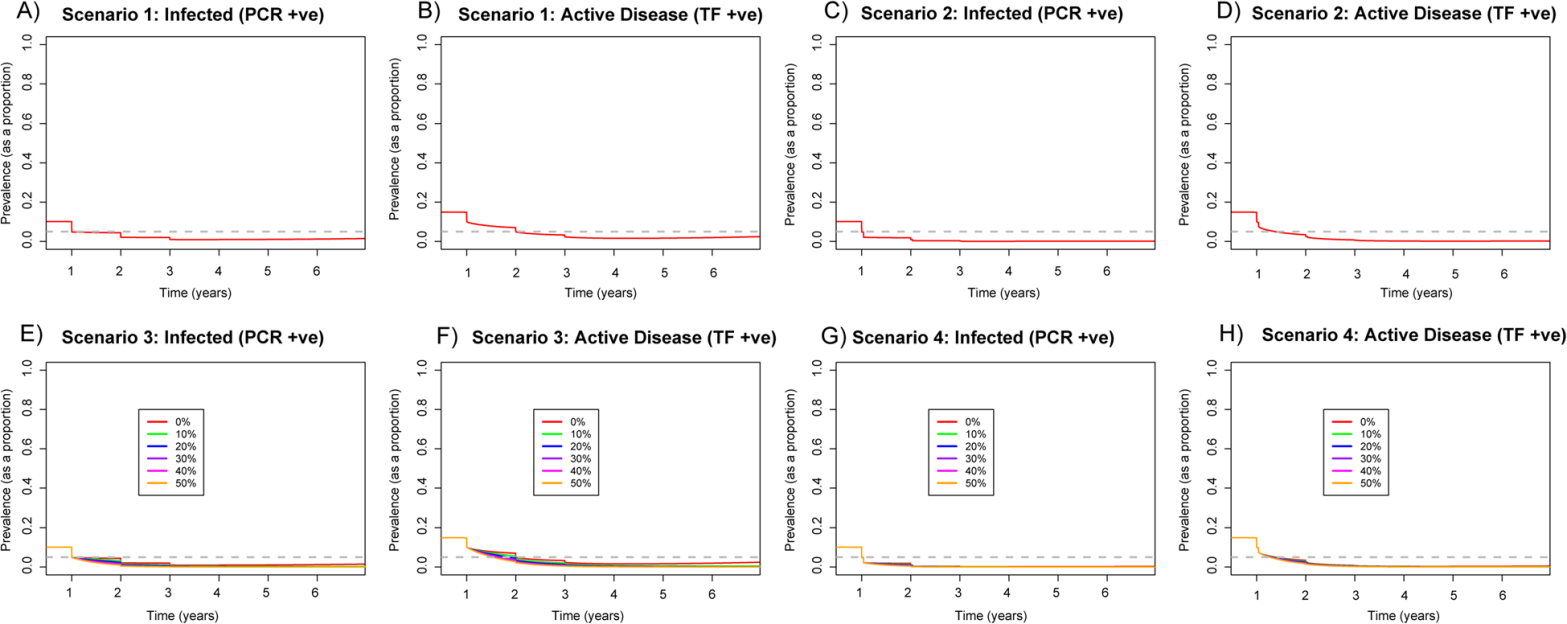
Prevalence of infection and active disease in 0–9-year olds when comparing single-dose annual MDA to double-dose annual MDA (conducted 2 weeks apart) within a hypoendemic community for 3 years. We assumed 80 % coverage, and 65 % and 85 % efficacy of the antibiotic for high and low bacterial loaders, respectively. Scenario one: **a** prevalence of infection and **b** active disease with single-dose annual MDA, with a 0 % decline in *β*. Scenario two: **c** prevalence of infection and **d** active disease with double-dose annual MDA applied 2 weeks apart, with a 0 % decline in *β*. Scenario three: **e** prevalence of infection and **f** active disease with one annual round of MDA. Scenario four: **g** prevalence of infection and **h** active disease with double-dose annual MDA applied 2 weeks apart. While also modelling an instantaneous drop in *β*, we present a range of reductions in *β. Different coloured lines* represent different percentage declines in the value of *β*, ranging from 0–50 %. *Grey dashed line* indicates 5 % prevalence, where < 5 % prevalence of active disease is the GET 2020 target level

After the first year of treatment with a single-dose MDA, in a hypoendemic setting the prevalence of active disease was not reduced to 5 % (Fig. 4a). While for scenario two, after the first year of treatment prevalence of active disease dropped to the target level of < 5 % and remained so 3 years after treatment ceased (Fig. 4d).

### MDA and transmission reduction through enhanced F&E

Transmission reduction in combination with MDA had a larger impact on prevalence of infection and active disease in the hyperendemic community, in comparison to MDA alone. For scenario three, at least 30 % transmission reduction was required in addition to MDA (Fig. 2e, f). While in scenario four (Fig. 2g, h), a reduction of greater than 10 % was sufficient to eliminate infection.

For the mesoendemic community, a 20 % decrease in transmission produced a comparable level of final prevalence across the 6-year period with scenarios three and four (Fig. 3e–h). With a 20 % reduction in transmission in both scenarios, no re-emergence of infection was observed (Fig. 3e–h). However, with scenario four, it may be possible to eliminate infection within 2 years.

MDA and transmission reduction measures resulted in large reductions in prevalence (Fig. 4e–h) in the hypoendemic setting. When *β* was reduced by at least 10 %, infection was successfully eliminated under both MDA distribution strategies, although infection was eliminated more quickly from the community under scenario four (Fig. 4e–h).

### Biannual treatment at 6-monthly intervals vs two doses 2 weeks apart annually for a hyperendemic community

A more dramatic reduction in the prevalence of infection and active disease was projected to be achieved with the annual double-dose treatment strategy (scenario two) in comparison to single-dose treatment at 6-monthly intervals (Additional file 1: Figure S3). After 2 years of treatment, prevalence of infection was reduced to 5 % for scenario two, and this reduction in prevalence was not achieved until 4 years after treatment was applied at 6-monthly intervals (Additional file 1: Figure S3). With an instantaneous drop in the transmission rate of 10 %, infection was eliminated within the community after six rounds of treatment with scenario four (Additional file 1: Figure S3g); however, it required ten rounds with 6-monthly treatment (Additional file 1: Figure S3e). Although, in both scenarios infection re-emerged in the community if transmission was reduced by only 10 % (Additional file 1: Figure S3e–h).

### Sensitivity analysis of the variation in coverage level

Across all transmission settings, as coverage and efficacy reduced, the effect of all interventions was reduced. For the hyperendemic community, if coverage was 60 % neither antibiotic distribution strategy was sufficient to control transmission and very high levels of transmission reduction were required to eliminate infection (Additional file 1: Figure S7–S9a–h). If coverage was increased to 70 %, MDA alone was not sufficient to maintain infection control in the hyperendemic community (Additional file 1: Figure S10a–d). However, if coverage was increased to 95 %, a steady decline in transmission was observed for all settings and scenarios (Additional file 1: Figure S13–S15a–h). Although, single-dose annual MDA showed limited impact on transmission reduction in the absence of enhanced F&E, particularly for hyperendemic communities.

### Sensitivity analysis of the variation in antibiotic efficacy

Assuming a baseline level of coverage (80 %), as efficacy of treatment reduced, the impact of MDA alone was also reduced. At the lowest assumed efficacy of 40 % and 70 % for high and low bacterial loaders, respectively, MDA alone had no long-term impact in any transmission setting, and enhanced F&E was also needed to reduce transmission (Additional file 1: Figure S16, S18, S20a–d). However, under all scenarios the percentage reduction in transmission through enhanced F&E required to control transmission was lower with the proposed double-dose strategy (Additional file 1: Figure S16, S18, S20e–h). As the assumed efficacy of treatment increased, the success of each intervention to reduce transmission improved (Additional file 1: Figure S17, S19, S21a–h). However, single-dose annual MDA alone was not sufficient to control infection within the hyperendemic community, and signs of re-emergence were apparent with the double-dose strategy, in the absence of enhanced F&E (Additional file 1: Figure S17).

## Discussion

This study explored the projected effectiveness of different antibiotic distribution strategies and levels of transmission reduction on prevalence of infection and disease in three different transmission settings, with the ultimate aim of achieving the GET 2020 goal of reducing prevalence of TF in 1–9-year olds to < 5 %. It has previously been demonstrated that the single-dose efficacy of azithromycin treatment is not 100 %, particularly for those with high bacterial loads [8, 10]. In the proposed double-dose strategy, in cases where the infection does not resolve, the first dose of antibiotic treatment reduces an individual’s bacterial load, thus when they are re-treated 2 weeks after the first dose, they have a higher probability of resolving their infection following the second dose. The implementation of this new strategy may also help to increase the overall level of coverage achieved across the two rounds, due to increased awareness, along with catching individuals who may have missed the first round of treatment. Although the number of antibiotic doses required initially would be higher than when single-round annual MDA is implemented, it is likely that the total number of doses required for long-term control would be fewer under the double-dose strategy as the treatment programme would need to run for a shorter time period.

To date, very few clinical studies have explicitly monitored the impact of water, sanitation and hygiene (WASH) or F&E on human behaviours; nor have they attempted to quantify the possible impact of these interventions on trachoma transmission within a clinical trails setting. Further to this, these interventions have, to our knowledge, not been previously assessed in the trachoma mathematical modelling literature. A key feature of the proposed clinical trial is the enhanced F&E component. This component seeks to draw ethnographic information on human behaviours and practices to identify key routes of trachoma transmission within the community, facilitating the formulation and development of highly specific and targeted F&E interventions, which aim to directly reduce trachoma transmission within the community. This will for the first time allow estimates of highly targeted transmission reduction measures for trachoma to be made.

We acknowledge that particularly for single-dose annual MDA, the rates of infection rebound in the model following treatment are generally higher than those observed in the field [5, 18, 19]. However, high variance in the number of cases reported following several rounds of MDA is cited [18]. Furthermore, many antibiotic treatment studies may have an F&E component, which is not always explicitly monitored or quantified. Therefore, we may expect an underlying degree of transmission reduction in these trials as well. As such, we may expect findings from trials to look like the simulations which model MDA with a small amount of transmission reduction. Indeed, we see that the output from these simulations show a much slower rebound effect.

We found that the GET 2020 goal of reducing prevalence of TF in children aged 1–9 years old to < 5 % may be achievable with the interventions explored for a number of transmission settings; however, monitoring will be needed (and is recommended) to check that the prevalence remains below the elimination threshold, as our results suggest that in the absence of transmission reduction interventions, infection may re-emerge. This observation is supported by MDA clinical trial data [5, 18, 19]. One recent theoretical modelling study suggested that if a community experienced 10 annual rounds of MDA and rapid transmission reduction, shifts in population immunity may be observed resulting in short-term increases in R0 [20]. However, the modelling results presented here suggest that with a smaller number of annual rounds of MDA and transmission reduction through F&E it is possible to eliminate infection in all transmission settings.

For hyperendemic communities, the new double-dose treatment regime may have a large impact on reducing prevalence of infection, in comparison to single-dose annual MDA. With a reduction of greater than 10 % in the transmission parameter *β* through enhanced F&E, it may be possible to control infection in hyperendemic communities. The annual double-dose strategy was projected to reduce prevalence of infection and disease more quickly than biannual treatment at 6-monthly intervals. However, as coverage and treatment efficacy reduced, the projected effect of impact was also reduced. For example, in the hyperendemic community, if coverage was only 60 %, neither strategy in the absence of enhanced F&E was sufficient to lower infection. This reinforces the importance of attaining high levels of treatment coverage to successfully control infection.

For hypo- and mesoendemic settings, the double-dose distribution strategy was projected to provide an advantage in helping to achieve the GET 2020 goal of reducing the prevalence of active disease in 1–9-year olds. The double-dose strategy reduced prevalence of infection more quickly in comparison to annual MDA, suggesting that it may help to maintain a lower prevalence of infection and active disease for longer periods. However, when efficacy of treatment and coverage level reduced, infection may be more likely to remerge.

Our study has a number of limitations. Firstly, we have assessed the impact of interventions using a deterministic mathematical model, which at high levels of transmission intensity is reasonable. However, as transmission reduces our model does not allow for the stochastic fade-out of infection that may occur at low levels of prevalence and transmission [21–23]. As such, we may have overestimated the effort required to control infection at lower levels of transmission intensity. Equally, re-emergence of infection at very low transmission intensity may only be an artefact of the deterministic model structure used here. However, it is also important to consider that stochastic re-emergence of infection within communities may occur. Secondly, the quantitative impact of enhanced F&E remains poorly understood; therefore, we have modelled this effect through direct reductions in the transmission rate parameter *β*. However, these interventions may act through indirect means not explicitly accounted for in the model. We do not know what these levels of transmission reduction would correspond to in terms of F&E control measures, nor do we know whether F&E would be equally accessed by all members of the population, though this seems a reasonable assumption for a model. Additionally, the reduction in transmission may not be instantaneous, but more gradual as hygiene and sanitation conditions improve over time. However, sensitivity analysis to this assumption suggested that it had relatively little impact in the meso- and hypoendemic settings (Additional file 1: Figure S35–S36). For hyperendemic communities, year-on-year reductions in infection prevalence were slightly lower when *β* declined more gradually over the 5-year period. However, as the total level of transmission reduction in the community achieved was the same in both situations, the results at the end of the intervention period were the same. Here, 10–20 % reduction in transmission was sufficient to eliminate infection with the double-dose treatment strategy (Fig. 2 and Additional file 1: Figure S34).

Thirdly, while we have assessed the impact of our different intervention scenarios across a range of coverage levels, it is possible that coverage may vary between years of intervention implementation—this may impact long-term trends in prevalence. Fourthly, as with many modelling studies we have made a number of simplifying assumptions, including assuming treatment efficacy was the same for all individuals who were low loader and those who were high. In reality, it is likely that efficacy between all individuals will vary to some extent. However, capturing the heterogeneity in response to treatment through high and low bacterial loads is one further step in the development of biologically plausible models. Further to this, we have assumed the primary mechanisms underlying the re-emergence of infection within the community are due to the failure of one antibiotic dose to fully clear infection in those who have high bacterial loads; however, the picture is likely to be more complex. We have assumed that individuals are missed from treatment at random, though there may be small groups of people or households who are systematically not treated, resulting in continuous transmission within these groups. However, ongoing transmission within the household or the community is to some extent accounted for in the re-emergence of infection within the community under certain scenarios.

In addition, for a given level of treatment efficacy we have assumed that for low loaders infection is fully cleared. However, the microbial cure rate with azithromycin for urogenital chlamydia is estimated to be 97–98 % [24]. This ‘latent chlamydia’ is believed to not be susceptible to treatment, and is reported to be the reason behind the imperfect cure rate for urogenital chlamydia. This observation may also extend to ocular chlamydia, but to our knowledge, data to support this in the ocular context remains limited. However, if there is a background level of latent chlamydia in the community that is untreatable, the rate of infection re-bound within MDA-treated communities may be higher than currently modelled here. The ability of individuals with latent chlamydial infection to act as a reservoir source of trachoma infection within the community remains unknown and warrants further investigation.

We have assumed that individuals with active disease are immune to re-infection, although a small amount of clinical data has suggested that this might not be true. However, very limited quantifiable information is available to parameterise this. Nevertheless, sensitivity analysis to this assumption suggested that an increase in the susceptibility to re-infection in the AD state could lead to a larger impact of the modelled interventions for both the single- and double-dose treatment regimens across all transmission settings (Additional file 1: Figure S37–S39). Lastly, we have assumed that immunity to infection is acquired exponentially, and high bacterial loads fall after the first ten infections. However, immunity to infection may take a different functional form, and it may require more or less infections to qualify as a high or low bacterial loader. A recent study in Kongwa, Tanzania, suggested that bacterial load prior to MDA was not predictive of continued infection in individuals following MDA [25]. However, the authors acknowledge that this is not consistent with their previous findings, and that the calculation of infection load between the two studies limits their analysis [25]. We feel that two published studies, which present conflicting results, do not provide sufficient evidence to refute the hypothesis evaluated in this article. Moreover, there is also support in the urogenital literature to suggest that organism load may be associated with treatment failure [26–28], highlighting that the role of bacterial load and its association with treatment failure is, at this point, not clear-cut.

## Conclusions

Despite a number of limitations, we have assessed the impact of our findings across a number of key assumptions, including assumptions relating to the variation in the number of infections that are high load and when immunity plateaus. Our results demonstrate the possible impact the new antibiotic distribution strategy may have in helping to reduce infection and disease, particularly in high prevalence communities to help achieve the GET 2020 goal of eliminating trachoma as a public health problem, and for some regions may help accelerate the timeline to elimination. However, in high transmission settings, MDA alone without sustained transmission reduction through enhanced F&E is likely to lead to re-emergence of infection within the community. The logistics of treating twice within a short time period means that fewer resources would be required than if treatment was conducted at 6-monthly intervals. Therefore, the projected impact this alternative antibiotic distribution strategy and transmission reduction measures may have provides a clear rationale for this strategy to be tested in a clinical trials setting. It would be expected that the trial would take a minimum of 3 years, with first reporting at year one.

## Additional file

Additional file 1: Additional detail on modelling methods and sensitivity analyses. (PDF 3614 kb)

## Abbreviations

E: environmental improvements
F: facial cleanliness
GET 2020: Alliance for the Global Elimination of Trachoma by the year 2020
MDA: mass drug administration
NPI: non-pharmaceutical intervention
SAFE: surgery, antibiotics, facial cleanliness and environmental improvements
SIS: susceptible, infected, susceptible
TF: trachomatous inflammation, follicular
TT: trachomatous trichiasis
WASH: water, sanitation and hygiene
WHO: World Health Organization
