# Supplementary material for: Enhanced antibiotic distribution strategies and the potential impact of facial cleanliness and environmental improvements for the sustained control of trachoma: a modelling study
Source: BMC Med. 2016 May 19;14:71. doi: 10.1186/s12916-016-0614-6 (PMC4872360; doi:10.1186/s12916-016-0614-6)
Supplement: Additional file 1: — Additional detail on modelling methods and sensitivity analyses. (PDF 3614 kb) [file 12916_2016_614_MOESM1_ESM.pdf]

Supplementary material. Enhanced antibiotic distribution strategies and the potential impact of Facial cleanliness and Environmental improvements for the sustained control of trachoma: a modelling study

Mathematical model of trachoma transmission

A schematic of the model structure is provided in Figure 1.

As individuals become infected, recover from infection and then recover from active disease, they move up along a ladder of infection [1, 2] through the susceptible (S_i), infected (I_i), infected and infectious (AI_i) and then to active disease only (AD_i) compartments, where each compartment is connected to the next. Discretised versions of the following continuous (in age and time) partial differential equations were used to describe the flow from one compartment to another in the simulation.

Individuals in the susceptible (*S*) state are susceptible to infection, while individuals in the infected (*I*) state are infected but not infectious, nor do they have active disease. Those in the active infection (*AI*) state are infectious and have active disease and are temporarily immune to re-infection, while those in active disease (*AD*) are still experiencing active disease but do not have an active infection, and are also temporarily immune to re-infection. We model 3 age classes: those aged zero - nine years old (the WHO key indicator group), 10 - 14 years old and 15 + years. All results (except for the impact on sequelae) are plotted only for one - nine year olds. We denote age class by *a*, and the number of infections experienced as *i*. Parameter values for all three transmission settings are provided in Table S1. Following infection in the *S* state, individuals progress into the *I* class at a rate λ, this rate of removal from *S* to *I* is independent of infection history, hence an individual's susceptibility to re-infection is independent of their previous infection. Individuals progress out of *I* at rate γ_i to the *AI* class where they are infectious and also present with active disease; they progress out at a rate σ_i and hence recover from infection. Here the rate of recovery from infection does depend on the number of previous infections. Individuals recover from *AD* at a rate ζ_i, which also depends on the number of previous infections experienced.

$$\frac{\partial S_i}{\partial t}+\frac{\partial S_i}{\partial a}=-\lambda_{(a)}S_i(a,t)-\mu_{(a)}S_i(a,t)+\zeta_{i-1}AD_{i-1}(a,t)+\mu_{(a)}\delta(i=1)$$
$$\frac{\partial I_i}{\partial t}+\frac{\partial I_i}{\partial a}=\lambda_{(a)}S_i(a,t)-\mu_{(a)}I_i(a,t)-\gamma_i I_i(a,t)$$
$$\frac{\partial AI_i}{\partial t}+\frac{\partial AI_i}{\partial a}=\gamma_i I_i(a,t)-\mu_{(a)}AI_i(a,t)-\sigma_i AI_i(a,t)$$
$$\frac{\partial AD_i}{\partial t}+\frac{\partial AD_i}{\partial a}=\sigma_i AI_i(a,t)-\mu_{(a)}AD_i(a,t)-\zeta_i AD_i(a,t)$$

Where λ is defined as:

$$\lambda_{(a)}=\int_{a'}w(a,a')\left(\beta\left(\frac{\sum_j \rho_j AI_j(a',t)}{\sum_j N_j(a',t)}\right)+v_2\left(\frac{\sum_j \rho_j AI_j(a',t)}{\sum_j N_j(a',t)}\right)^{v+1}\right)da'$$

It has recently been suggested that the dynamics of trachoma may facilitate elimination [3]. Here, we assume that individuals are exposed to a linear and non-linear force of infection (as previously modelled) [3].

ρ_j is the infectivity of an individual in compartment AI_j. γ is the rate at which infected individuals I_i progress to becoming infected and infectious, σ_i is the rate at which IA_i individuals recover from infection, ζ_i is the rate

at which AD_i individuals recover from active disease only. $w(a, a')$ is a mixing matrix which contains information on the rate of mixing between individuals of age group a and a' [4].

$$w(a, a') = \varepsilon\, \delta_{a,a'} + (1 - \epsilon) \frac{N_{a'}}{\sum_{a'} N_{a'}}$$

$\delta_{a,a'}$ is the Kronecker Delta. $N_{a'}$ is the number of individuals of age a' in the population and ε indicates the degree of mixing assortativity, which can range between 0 (random age mixing) and 1 (fully assortative).

Rate of recovery

The per individual rate of recovery from infection σ_i (measured as a rate per year) from infection *i* the rate of recovery is assumed to change as an exponential function of *i* that begins at a rate of σ_1 (recovery from the first infection) and rises to a maximum rate σ_{100} where no greater rate of recovery can be achieved after this point.[2]

$$\sigma_i = (\sigma_1 - \sigma_{100})\exp[-\omega\,(i-1)] + \sigma_{100}$$

We also model the per individual rate of recovery from active disease only ζ_i (measured as a rate per year) from infection *i* the rate of recovery is assumed to change as an exponential function of *i* that begins at a rate of ζ_1 (recovery from the first infection) and rises to a maximum rate ζ_{100}. We assume the rate of change of the recovery rate per infection (ω) is the same for recovery from infection and active disease.

$$\zeta_i = (\zeta_1 - \zeta_{100})\exp[-\omega\,(i-1)] + \zeta_{100}$$

Values of these parameters are provided in Table S1.

Infectivity

We assumed the infectivity of an individual (ρ_j) was proportional to the log of their bacterial load l_i, which was a function of the number of previous infections experienced by each individual, a trend that is in agreement with the data from trachoma endemic communities in which the bacterial load decreases with age [5, 6]. We assumed that an individual's load decreased with an increasing number of infections experienced. We assumed a linear decline in the log of the bacterial load. This function also saturated after 100 infections had been experienced.

Treatment of individuals

All individuals in the *I* and *AI* states can be treated effectively with antibiotics, and antibiotics do not treat those in the *AD* class. We assume a differential efficacy of treatment for individuals who have high and low bacterial loads. For individuals who have experienced 10% of the maximum number of infections, we assumed that the immune response was not strong enough to control infection well and therefore these individuals had a high bacterial load. This assumption is supported by clinical data from Tanzania and Gambia, which shows that infections with high bacterial loads were more commonly found in children than adults, hence we assume that young children have experienced fewer infections than adults [5, 7, 8]. Following the first 10% of infections, infected individuals had a lower bacterial load, and efficacy of treatment was higher. Baseline efficacy parameters are provided in Table S1.

We modelled treatment so that individuals in I_i who are treated successfully return to the S_i state they were in before they were infected. However, for those in AI_i, if they were treated successfully we assumed that they then moved into S_{i+1} state. A schematic of this movement is presented in Figure S1.

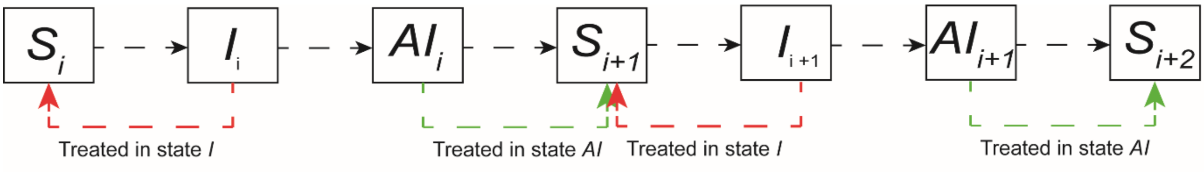

Additional file 1: Figure S1. A schematic of how infected individuals move along the infection ladder when successfully treated. Individuals who are infected but not infectious when treated return to the S_i class they were in before they were infected (indicated by the red arrow), hence no immunity is acquired as a result of infection. For those in the AI_i class who are successfully treated they progress to the *i* +1 state (indicated by the green arrow) and are assumed to acquire immunity as a consequence of the infection they experienced.

Calculating the number of people treated at each time point

In scenarios 1 and 3 we calculated the number of people effectively treated (i.e. the number for whom bacterial load is cleared) in the high and low bacterial load groups as $c*e_{h/l}$, which is simply coverage multiplied by the efficacy of the antibiotic, dependent on whether an individual had a high or low bacterial load. The reason that this calculation was so simple was that, in these scenarios, a single round of antibiotic is being administered to the community.

It is more complicated to estimate the number of people effectively treated in two rounds of treatment, since the overall effectiveness of two rounds of treatment in the community depends upon: a) whether an individual was treated at both rounds i.e. did an individual receive two doses of antibiotic rather than one? b) did a twice-treated individual initially harbour a high or a low bacterial load? The second of these points is important because a single antibiotic dose for a high load individual will either clear the load, reduce the high load to a low bacterial load, or fail i.e. do nothing. Our model structure does not easily allow us to track those who have or have not been treated once, to allow us to apply different antibiotic efficacies on these different groups. We therefore perform a calculation that accounts for the effective coverage over two rounds but is applied *as if* it were a single treatment round. For high loaders in scenarios 2 and 4, this was calculated as:

$(c^2)e_h + (c^2)(1-e_h)0.5(e_h + e_l) + 2(c - c^2)e_h$

The first of these terms is simply the proportion of the population that clears a high load infection after a single treatment round, despite receiving two treatment rounds. The second term refers to the proportion of the population for whom the first round 'fails' but the second round succeeds in clearing infection. This group is made up of two subgroups: those for whom the first round leaves them in a high bacterial load state, and those for whom the high bacterial load has been reduced to a low one; these subgroups are modelled as being 50% each of the population that 'fails' the first round (failure rate after 1 round of treatment, according to West *et al* [6], was observed to be 10-15% of those high-loaders who were treated). The third term is the population proportion which receives only a single treatment round, of the two administered to the community (because they miss the second round i.e. they do not show up for treatment the second time around) but the single round is sufficient to clear their high bacterial load. Figure S2 illustrates the how we calculated the coverage level, and it assumes random treatment

For low loaders this was:

$(c^2)e_l + (c^2)(1 - e_l)e_l + 2(c - c^2)e_l$

Each of the three terms comprising this expression have analogous explanations to the terms in the high load expression above.

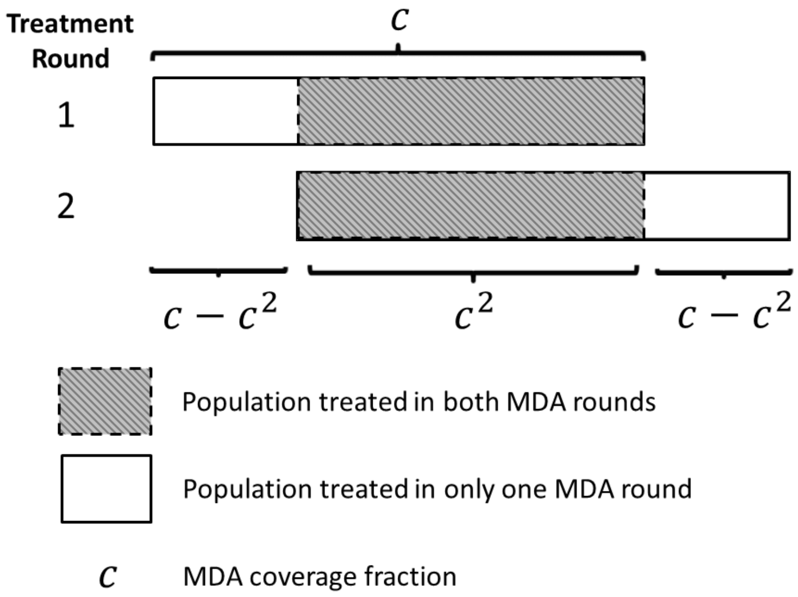

Additional file 1: Figure S2. Schematic illustration of the proportion of the modelled community that receives single or a double round of treatment. Here, each round of treatment has a coverage *c* and, because we assume that treatment is administered randomly, c^2 is the population proportion receiving two doses. The proportion receiving only one of the two doses is therefore $2(c - c^2)$.

Parameters

Additional file 1: Table S1. State variables, parameters definitions and values used in the model. Values indicated in brackets show the range of values used in the sensitivity analysis performed for coverage level and treatment efficacy. Where three numbers are listed for β and ϑ, they indicate the values used for hyper, meso and hypoendemic communities.

Name	Definition	Value	Units	Source
S_i	State variable			
I_i	State variable			
AI_i	State variable			
AD_i	State variable			
c	Coverage level of treatment	80% (60 – 95%)	Percentage	
e_l	Efficacy of antibiotic for low bacterial loaders	85% (70 – 90%)	Percentage	[9, 10]
e_h	Efficacy of antibiotic for high bacterial loaders	65% (45 – 70%)	Percentage	[9, 10]
ε	Degree of random mixing in the population	0.5	Proportion	[1, 2]
μ	Birth/death rate	1/60	Years^{-1}	
γ	Rate at which infected individuals become infectious	1/5	Days^{-1}	[11]
σ_1	Minimum rate of recovery from 1st infection	1/155	Days^{-1}	[11]
σ_{100}	Maximum rate of recovery from 100th infection	1/77	Days^{-1}	[11]
ζ_1	Minimum rate of recovery from active disease after 1st infection	1/209	Days^{-1}	[11]
ζ_{100}	Maximum rate of recovery from active disease after 100th infection	1/7	Days^{-1}	[11]
ρ	Infectivity of an individual proportional to the log of their bacterial load	Range from 0-1	Proportion	[2]
β	Transmission rate parameter	0.018, 0.011, 0.009	Days^{-1}	
v_2	Non-linear constant term	2.6	Number	[3]
v_1	Non-linear power term	0.8, 1 , 1	Number	[3]
ω	Rate of change of the recovery rate per infection	0.4	Infection^{-1}	[11]

N_infs	Maximum number of infections before immunity saturates	100 (50 – 200)	Number	
N_h	Number of infections that were classified as high load	10 (5 – 20)	Number	

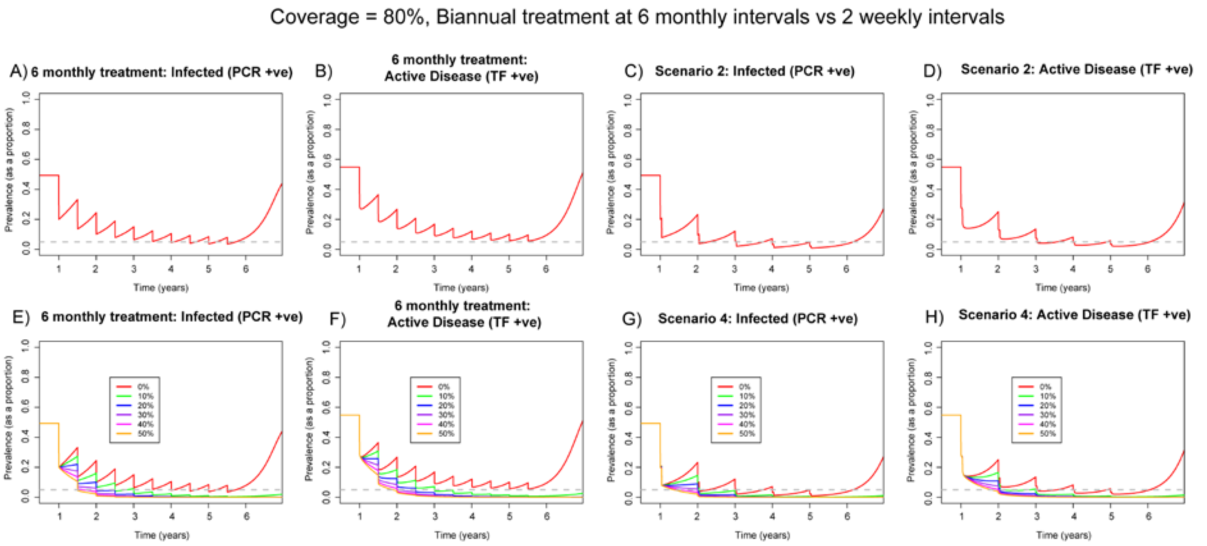

Additional file 1: Figure S3. Prevalence of infection and active disease in 0- 9 year olds when comparing biannual treatment at six-monthly intervals to double-dose annual MDA applied two weeks apart within a hyperendemic community for five years. Assuming 80% coverage and 65% and 85% efficacy of the antibiotic for high and low bacterial loaders respectively. A) prevalence of infection and B) active disease with biannual MDA at six-monthly intervals. C) prevalence of infection and D) active disease with double-dose annual MDA two weeks apart. E) prevalence of infection and F) active disease when biannual MDA is applied at six-monthly intervals and G) prevalence of infection and H) active disease with double-dose annual MDA two weeks apart while also modelling an instantaneous drop in β, we present a range of reductions in β through enhanced F&E. Different coloured lines represent different percentage declines in the value of β. Grey dashed line indicates 5% prevalence, where <5% prevalence of active disease is the GET 2020 target level.

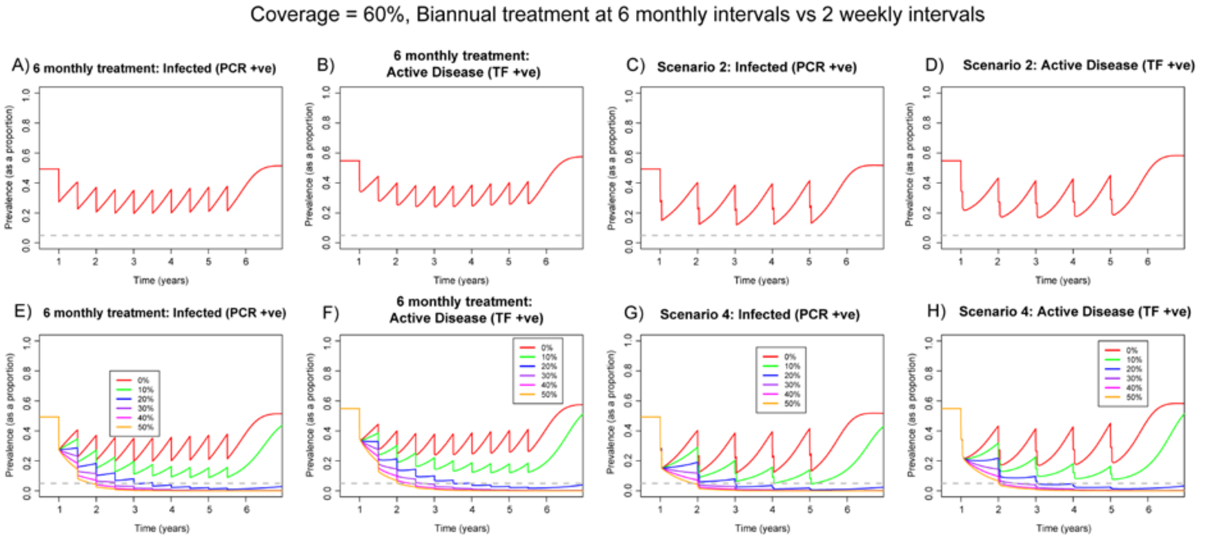

Additional file 1: Figure S4. Prevalence of infection and active disease in 0- 9 year olds when comparing biannual treatment at six-monthly intervals to double-dose annual MDA applied two weeks apart within a hyperendemic community for five years. Assuming 60% coverage and 65% and 85% efficacy of the antibiotic for high and low bacterial loaders respectively. A) prevalence of infection and B) active disease with biannual MDA at six-monthly intervals. C) prevalence of infection and D) active disease with double-dose annual MDA two weeks apart. E) prevalence of infection and F) active disease when biannual MDA is applied at six-monthly intervals and G) prevalence of infection and H) active disease with double-dose annual MDA two weeks apart, while also modelling an instantaneous drop in β, we present a range of reductions in beta. Different coloured lines represent different percentage declines in the value of β through enhanced F&E, ranging from 0% - 50%. Grey dashed line indicates 5% prevalence, where <5% prevalence of active disease is the GET 2020 target level.

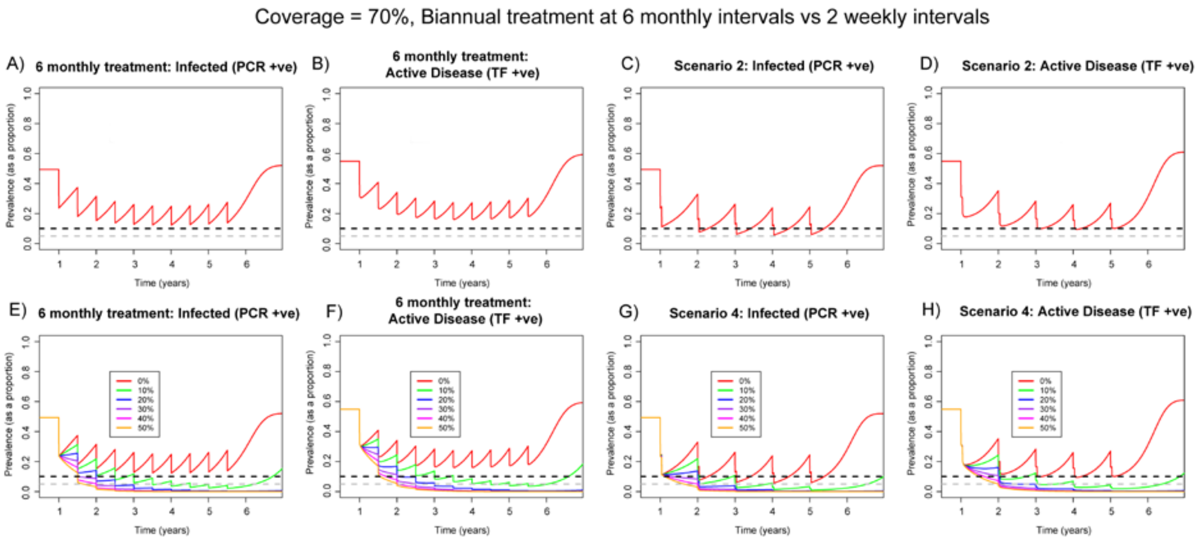

Additional file 1: Figure S5. Prevalence of infection and active disease in 0 - 9 year olds when comparing biannual treatment at six monthly intervals and biannual treatment two weeks apart annually within a hyperendemic community for five years. Assuming 70% coverage and 65% and 85% efficacy of the antibiotic for high and low bacterial loaders respectively. A) prevalence of infection and B) active disease with biannual MDA at 6 monthly intervals. C) prevalence of infection and D) active disease with double-dose annual MDA two weeks apart. E) prevalence of infection and F) active disease when biannual MDA is applied at six monthly intervals and G) prevalence of infection and H) active disease with double-dose annual MDA two weeks apart, while also modelling an instantaneous drop in β, we present a range of reductions in beta. Different coloured lines represent different percentage declines in the value of β through enhanced F&E, ranging from 0% - 50%. Grey dashed line indicates 5% prevalence, where <5% prevalence of active disease is the GET 2020 target level.

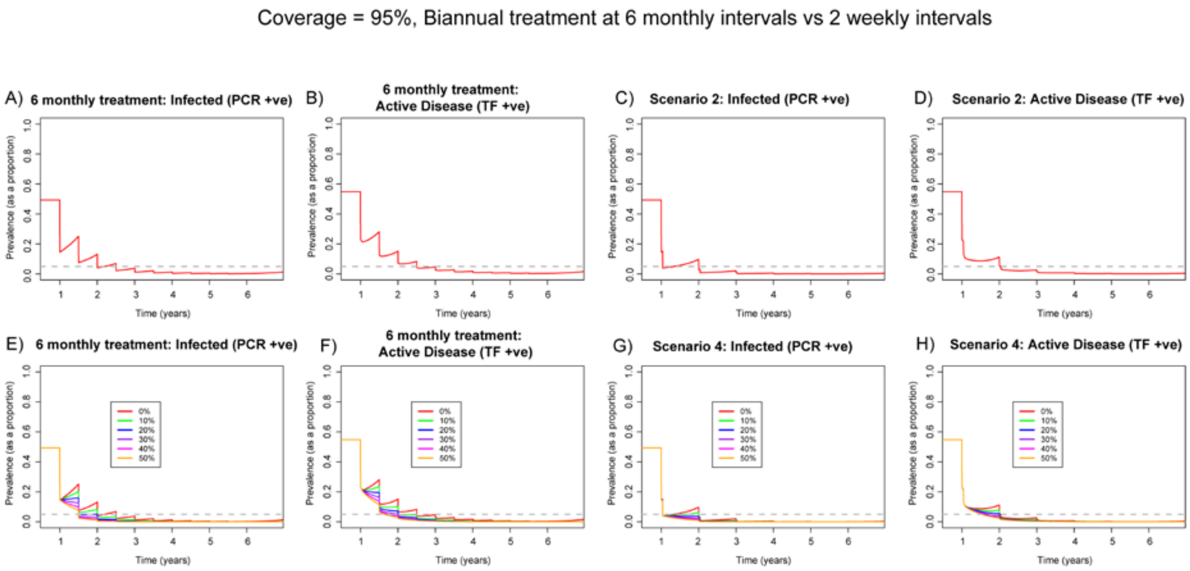

Additional file 1: Figure S6. Prevalence of infection and active disease in 0 - 9 year olds when comparing biannual treatment at six-monthly intervals and biannual treatment conducted two weeks apart within a hyperendemic community for five years. Assuming 95% coverage and 65% and 85% efficacy of the antibiotic for high and low bacterial loaders respectively. A) prevalence of infection and B) active disease with biannual MDA at 6 monthly intervals. C) prevalence of infection and D) active disease with double-dose annual MDA two weeks apart. E) prevalence of infection and F) active disease when biannual MDA is applied at six-monthly intervals and G) prevalence of infection and H) active disease with double-dose annual MDA two weeks apart, while also modelling an instantaneous drop in β, we present a range of reductions in beta. Different coloured lines represent different percentage declines in the value of β through enhanced F&E, ranging from 0% - 50%. Grey dashed line indicates 5% prevalence, where <5% prevalence of active disease is the GET 2020 target level.

Sensitivity analysis comparing biannual treatment with the two dose strategy

If coverage was 60%, MDA alone was insufficient to eliminate infection and even bring the prevalence of infection below 10% (Figure S4). Although, the short term reductions in prevalence were much greater with the double-dose treatment strategy in comparison to treatment at six-monthly intervals. If combined with MDA, transmission reduction needed to be at least 30% to successfully eliminate infection. If coverage was increased slightly to 70% (Figure S5) in the absence of any transmission reduction infection re-emerged within the community after each round of MDA. In both scenarios MDA with 20% transmission reduction was needed to eliminate infection within the community; however, prevalence of infection and disease declined more dramatically with two treatments two weeks apart. If coverage was 95%, infection was eliminated under both treatment regimes (Figure S6), however, following cessation of treatment a slow re-emergence of infection was observed with six-monthly treatment (Figure S6a&b). With 10% or more reduction in transmission infection was eliminated from the community under both treatment regimes, however infection was eliminated more quickly with the double-dose treatment strategy (Figure S6e-h).

Sensitivity of the baseline results to variation in coverage

MDA alone

Across all transmission settings control of infection was much more successful when a higher level of coverage was achieved. For the hyperendemic community when coverage was 60% a single dose or double-dose of annual MDA (scenario one) had limited impact and infection re-bounded to pre-treatment levels by the next treatment round (Figure S7a-d). In the mesoendemic community a slow re-emergence of infection was observed following the three rounds of MDA, however re-emergence occurred much more slowly in scenario two (Figure S8a-d). While in the hypoendemic community infection was still eliminated with 60% coverage, however it began to re-emergence following treatment cessation in scenario one but not scenario two (Figure S9a-d). If coverage increased to 95%, in the hyperendemic community a limited impact on the prevalence of infection was observed for scenario one, but infection was initially eliminated with scenario two following five years of treatment. For the mesoendmic community, for scenario one infection within the community was initially eliminated, but slowly re-emerged after treatment ended, this was not the case for scenario two. In the hypoendemic community infection as successfully eliminated from the community under both treatment scenarios with no evidence of re-emergence following three years of treatment cessation.

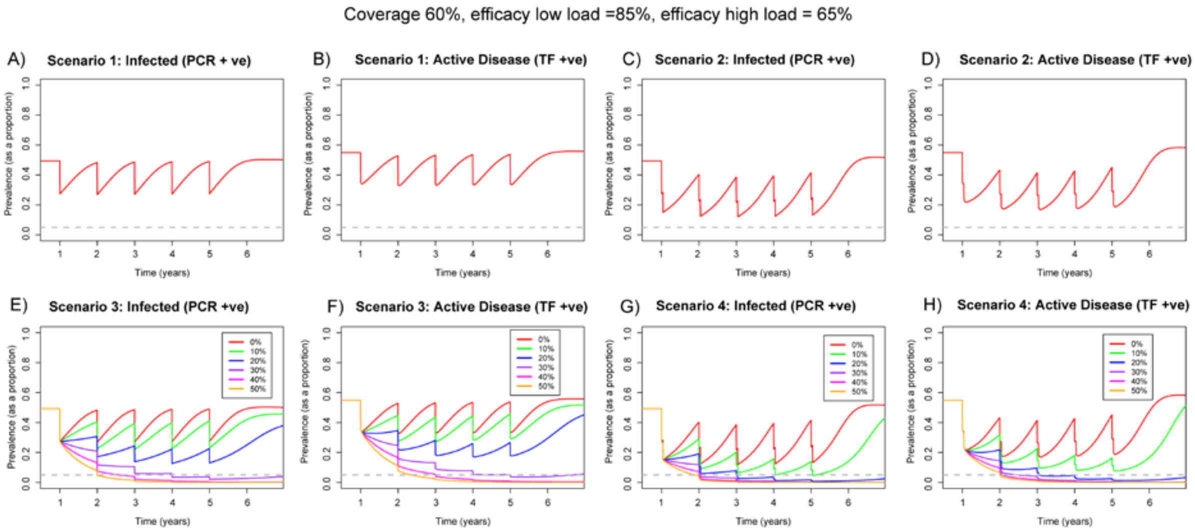

Additional file 1: Figure S7. Prevalence of infection and active disease in 0-9 year olds when comparing single-dose annual MDA to double-dose annual MDA (conducted two weeks apart) within a hyperendemic community for five years. Assuming 60% coverage and 65% and 85% efficacy of the antibiotic for high and low bacterial loaders respectively. A) prevalence of infection and B) active disease with single-dose annual MDA. C) prevalence of infection and D) active disease with double-dose annual MDA applied two weeks apart. E) prevalence of infection and F) active disease with single-dose annual MDA and G) prevalence of infection and H) active disease with double-dose annual MDA applied two weeks apart, while also modelling an instantaneous drop in β, we present a range of reductions in beta. Different coloured lines represent different percentage declines in the value of β through enhanced F&E, ranging from 0% - 50%. Grey dashed line indicates 5% prevalence, where <5% prevalence of active disease is the GET 2020 target level.

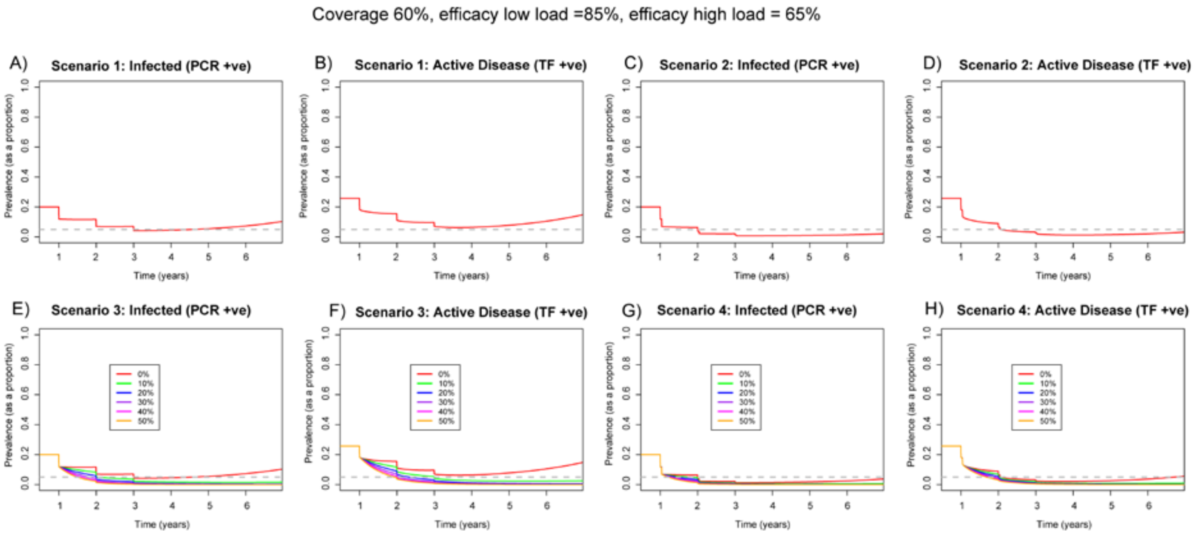

Additional file 1: Figure S8. Prevalence of infection and active disease in 0- 9 year olds when comparing single-dose annual MDA to double-dose annual MDA (conducted two weeks apart) within a mesoendemic community for three years. Assuming 60% coverage and 65% and 85% efficacy of the antibiotic for high and low bacterial loaders respectively A) prevalence of infection and B) active disease with single-dose annual MDA. C) prevalence of infection and D) active disease with double-dose annual MDA applied two weeks apart. E) prevalence of infection and F) active disease with single-dose annual MDA and G) prevalence of infection and H) active disease with double-dose annual MDA applied two weeks apart, while also modelling an instantaneous drop in β, we present a range of reductions in beta. Different coloured lines represent different percentage declines in the value of β through enhanced F&E, ranging from 0% - 50%. Grey dashed line indicates 5% prevalence, where <5% prevalence of active disease is the GET 2020 target level.

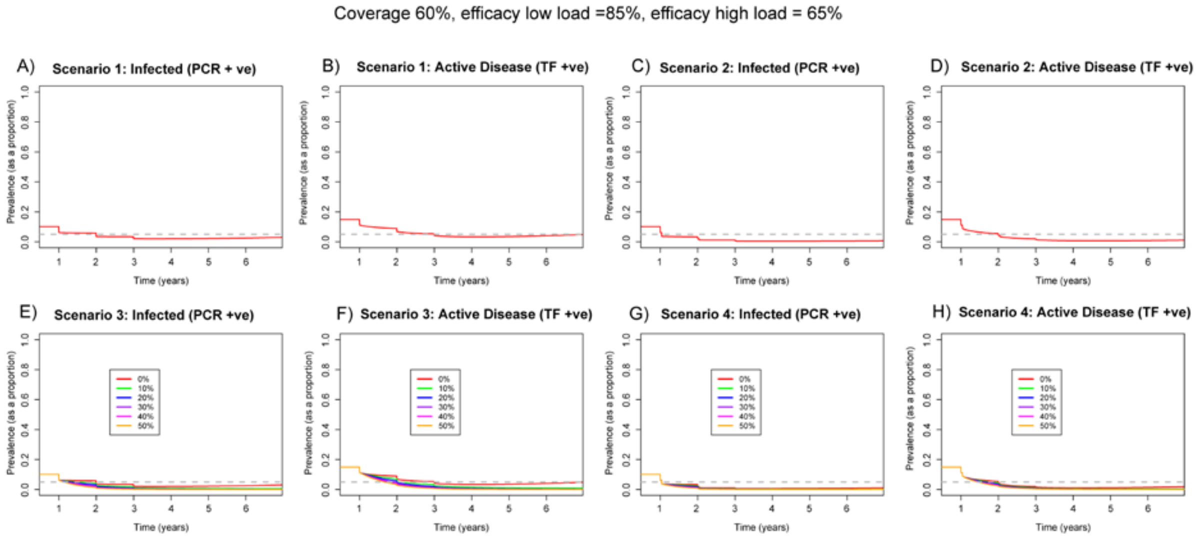

Additional file 1: Figure S9. Prevalence of infection and active disease in 0- 9 year olds when comparing single-dose annual MDA to double-dose annual MDA (conducted two weeks apart) within a hypoendemic community for three years. Assuming 60% coverage and 65% and 85% efficacy of the antibiotic for high and low bacterial loaders respectively. A) prevalence of infection and B) active disease with single-dose annual

MDA. C) prevalence of infection and D) active disease with double-dose annual MDA applied two weeks apart. E) prevalence of infection and F) active disease with single-dose annual MDA and G) prevalence of infection and H) active disease with double-dose annual MDA applied two weeks apart, while also modelling an instantaneous drop in β, we present a range of reductions in beta. Different coloured lines represent different percentage declines in the value of β through enhanced F&E, ranging from 0% - 50%. Grey dashed line indicates 5% prevalence, where <5% prevalence of active disease is the GET 2020 target level.

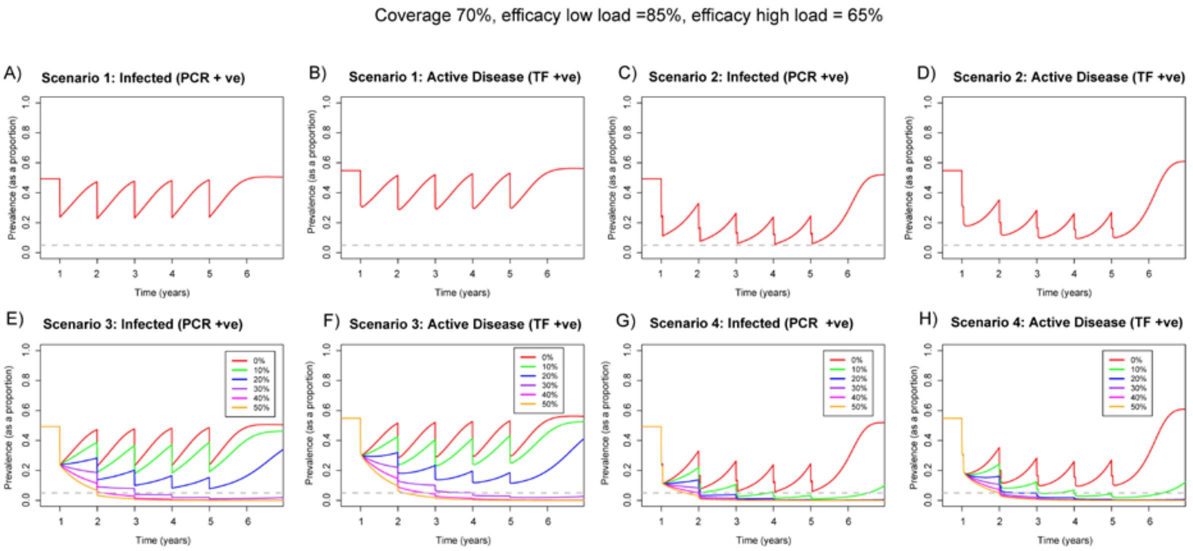

Additional file 1: Figure S10. Prevalence of infection and active disease in 0- 9 year olds when comparing single-dose annual MDA to double-dose annual MDA (conducted two weeks apart) within a hyperendemic community for five years. Assuming 70% coverage and 65% and 85% efficacy of the antibiotic for high and low bacterial loaders respectively. A) prevalence of infection and B) active disease with one annual round of MDA. C) prevalence of infection and D) active disease with two rounds of MDA applied two weeks apart annually. E) prevalence of infection and F) active disease with one annual round of MDA and G) prevalence of infection and H) active disease with two rounds of MDA applied two weeks apart annually while also modelling an instantaneous drop in β, we present a range of reductions in beta. Different coloured lines represent different percentage declines in the value of β through enhanced F&E, ranging from 0% - 50%. Grey dashed line indicates 5% prevalence, where <5% prevalence of active disease is the GET 2020 target level.

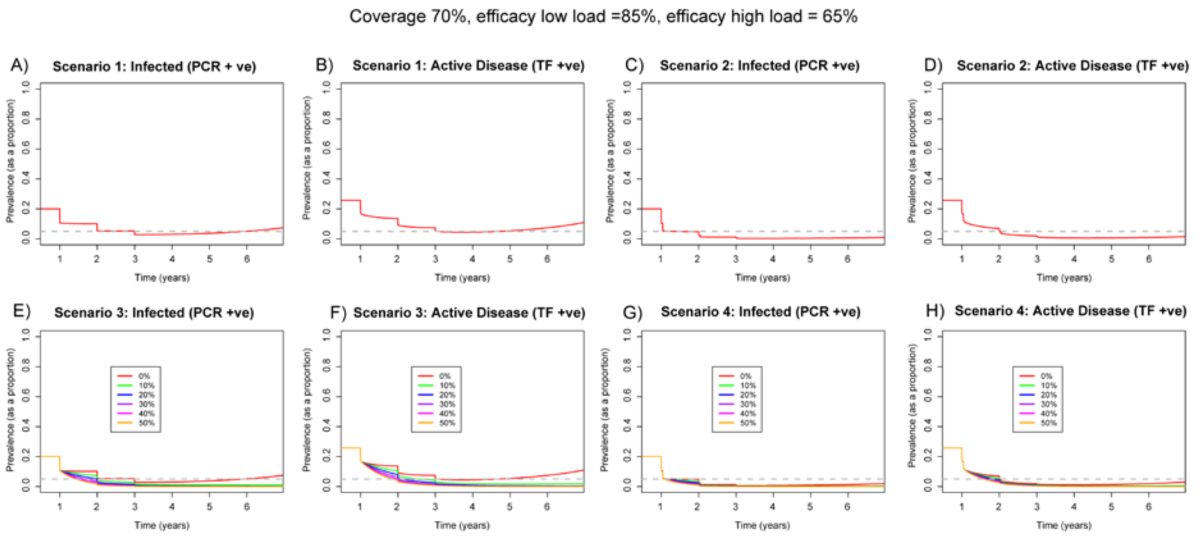

Additional file 1: Figure S11. Prevalence of infection and active disease in 0- 9 year olds when comparing single-dose annual MDA to double-dose annual MDA (conducted two weeks apart) within a mesoendemic community for three years. Assuming 70% coverage and 65% and 85% efficacy of the antibiotic for high and low bacterial loaders respectively. A) prevalence of infection and B) active disease with single-dose annual MDA. C) prevalence of infection and D) active disease with double-dose annual MDA applied two weeks apart. E) prevalence of infection and F) active disease with single-dose annual MDA and G) prevalence of infection and H) active disease with double-dose annual MDA applied two weeks apart, while also modelling an instantaneous drop in β, we present a range of reductions in β. Different coloured lines represent different percentage declines in the value of β through enhanced F&E, ranging from 0% - 50%. Black and grey dashed line indicates 10% and 5% prevalence respectively.

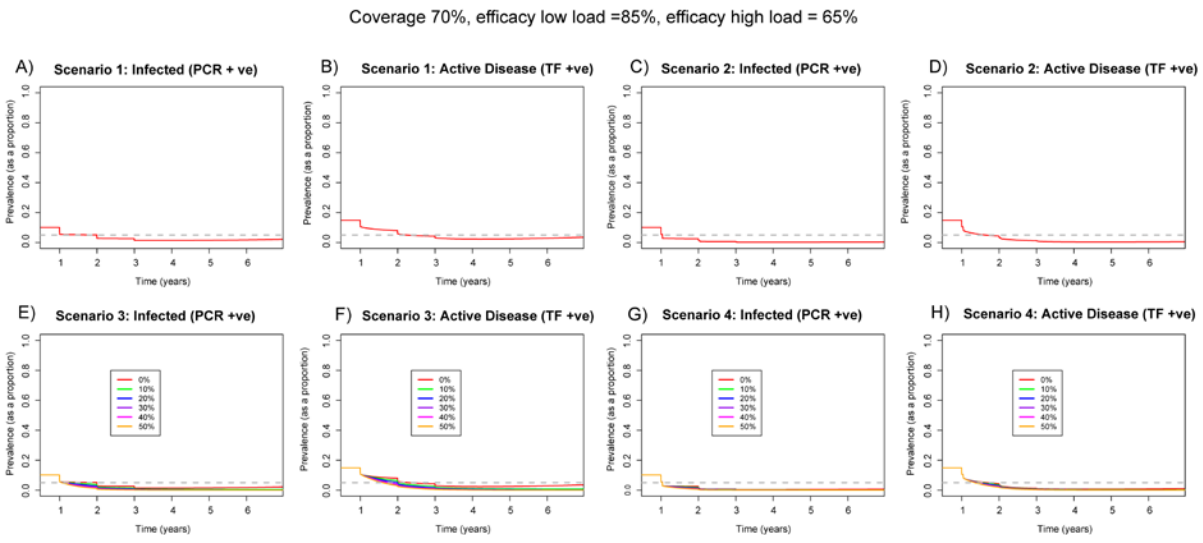

Additional file 1: Figure S12. Prevalence of infection and active disease in 0- 9 year olds when comparing single-dose annual MDA to double-dose annual MDA (conducted two weeks apart) within a hypoendemic community for three years. Assuming 70% coverage and 65% and 85% efficacy of the antibiotic for high and low bacterial loaders respectively. A) prevalence of infection and B) active disease with single-dose annual

MDA. C) prevalence of infection and D) active disease with double-dose annual MDA applied two weeks apart. E) prevalence of infection and F) active disease with single-dose annual MDA and G) prevalence of infection and H) active disease with double-dose annual MDA applied two weeks apart, while also modelling an instantaneous drop in β, we present a range of reductions in β. Different coloured lines represent different percentage declines in the value of β through enhanced F&E, ranging from 0% - 50%. Grey dashed line indicates 5% prevalence, where <5% prevalence of active disease is the GET 2020 target level.

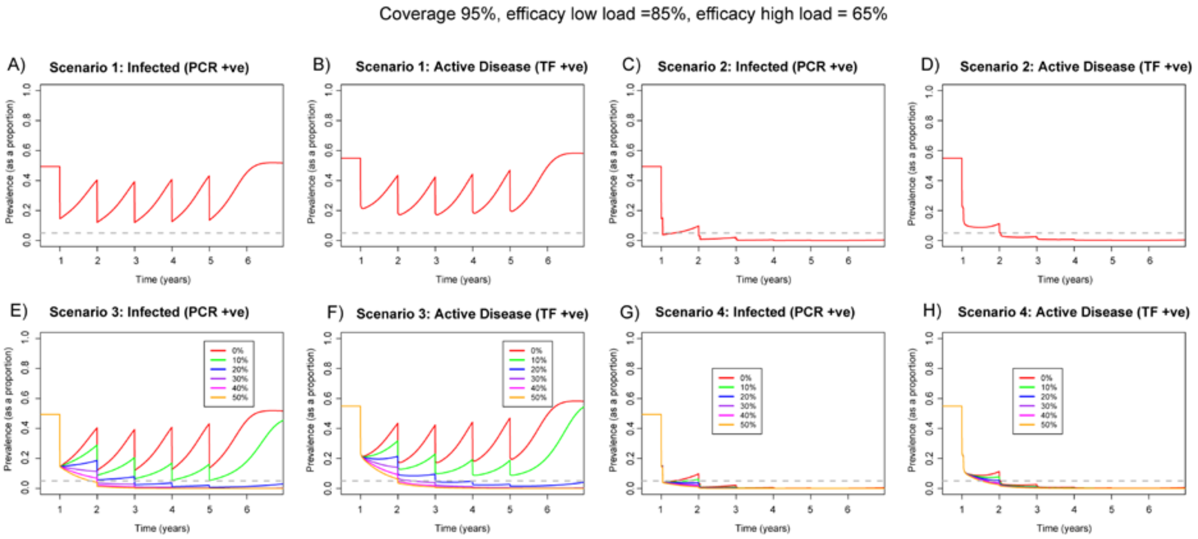

Additional file 1: Figure S13. Prevalence of infection and active disease in 0- 9 year olds when comparing single-dose annual MDA to double-dose annual MDA (conducted two weeks apart) within a hyperendemic community for five years. Assuming 95% coverage and 65% and 85% efficacy of the antibiotic for high and low bacterial loaders respectively. A) prevalence of infection and B) active disease with single-dose annual MDA. C) prevalence of infection and D) active disease with double-dose annual MDA applied two weeks apart. E) prevalence of infection and F) active disease with single-dose annual MDA and G) prevalence of infection and H) active disease with double-dose annual MDA applied two weeks apart, while also modelling an instantaneous drop in β, we present a range of reductions in beta. Different coloured lines represent different percentage declines in the value of β through enhanced F&E, ranging from 0% - 50%. Grey dashed line indicates 5% prevalence, where <5% prevalence of active disease is the GET 2020 target level.

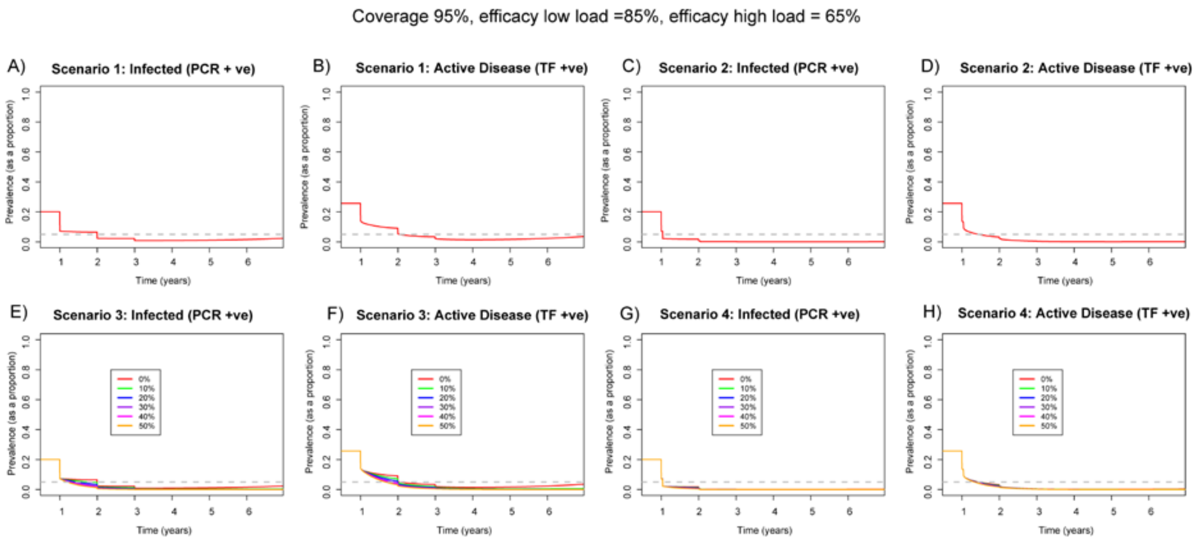

Additional file 1: Figure S1. Prevalence of infection and active disease in 0- 9 year olds when comparing single-dose annual MDA to double-dose annual MDA (conducted two weeks apart) within a mesoendemic community for three years. Assuming 95% coverage and 65% and 85% efficacy of the antibiotic for high and low bacterial loaders respectively. A) prevalence of infection and B) active disease with single-dose annual MDA. C) prevalence of infection and D) active disease with double-dose annual MDA applied two weeks apart. E) prevalence of infection and F) active disease with single-dose annual MDA and G) prevalence of infection and H) active disease with double-dose annual MDA applied two weeks apart, while also modelling an instantaneous drop in β, we present a range of reductions in beta. Different coloured lines represent different percentage declines in the value of β through enhanced F&E, ranging from 0% - 50%. Grey dashed line indicates 5% prevalence, where <5% prevalence of active disease is the GET 2020 target level.

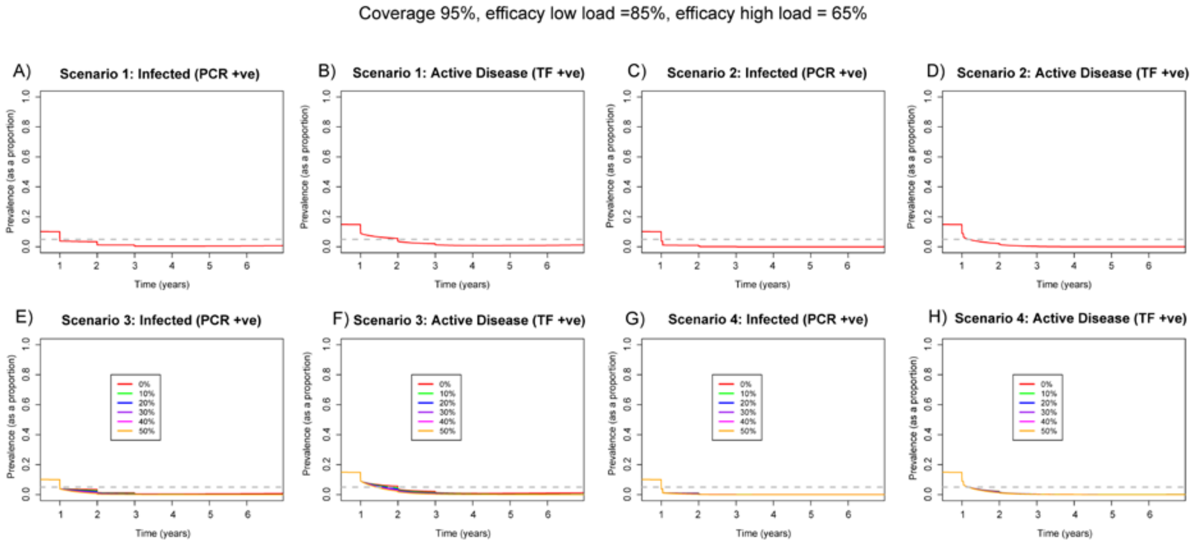

Additional file 1: Figure S15. Prevalence of infection and active disease in 0- 9 year olds when comparing single-dose annual MDA to double-dose annual MDA (conducted two weeks apart) within a hypoendemic community for three years. Assuming 95% coverage and 65% and 85% efficacy of the antibiotic for high and low bacterial loaders respectively. A) prevalence of infection and B) active disease with single-dose annual MDA. C) prevalence of infection and D) active disease with double-dose annual MDA applied two weeks apart.

E) prevalence of infection and F) active disease with single-dose annual MDA and G) prevalence of infection and H) active disease with double-dose annual MDA applied two weeks apart, while also modelling an instantaneous drop in β, we present a range of reductions in beta. Different coloured lines represent different percentage declines in the value of β through enhanced F&E, ranging from 0% - 50%. Grey dashed line indicates 5% prevalence, where <5% prevalence of active disease is the GET 2020 target level.

MDA and transmission reduction

As the coverage level reduced, a greater reduction in the transmission rate was needed in order to eliminate infection and prevent re-emergence, but the reduction required was always higher for scenario one in comparison to scenario two. When coverage was 60% in the hyperendemic community (Figure S7), at least a 40% reduction in transmission was needed to control infection for scenario three, while for scenario four a maximum of 30% was needed (Figure S7g&h). As coverage increased to 95%, a 30% reduction in transmission was sufficient to control infection for scenario three (Figure S13e&f), however no transmission reduction was needed in scenario four to eliminate and control infection (Figure S13g&h). For the mesoendemic community when coverage was 60%, a 20% or more reduction in transmission was required to control infection (Figure S8e&f), while in scenario four a reduction of 10% was sufficient (Figure S8g&h). However, if coverage was increased to 95% only a reduction in β of 10% was needed for scenarios three and four (Figure S14e-h). For the hypoendemic community even when coverage was 60%, a transmission reduction of 20% and 10% or more was sufficient to control infection in both treatment distribution scenarios three (Figure S9e&f) and four (Figure S9g&h) respectively. As coverage increased to 95% a reduction in the transmission rate of at least 10% was needed for scenario three however it was not necessary for scenario four (Figure S15e-h).

Sensitivity of the results to variation in treatment efficacy

When efficacy of treatment was assumed to be low for high and low bacterial loaders (40% vs 70% for each load group respectively), MDA alone was insufficient to reduce long-term infection prevalence in the hyperendemic community (Figure S16a-d), thus a greater level of transmission reduction was needed to control infection, for scenario three an instantaneous drop in transmission of 40% was needed (Figure S16e&f). While for scenario four a reduction of at least 30% was needed. If efficacy was higher than the baseline, scenario one had no long-term impact on reducing transmission (Figure S17a&b), in contrast, for scenario two infection was nearly eliminated from the community after five rounds of MDA (Figure S17c&d). Therefore, for scenario four only 10% reduction in transmission was required to control infection (Figure S17g&h), however for scenario three at least a 30% reduction in transmission was needed (Figure S17e&f).

For the mesoendemic community if efficacy of treatment was assumed to be low, MDA alone under either distribution strategy could not eliminate infection, indeed for scenario one after three rounds of MDA infection prevalence was not even reduced to 5% (Figure S18). In scenarios three and four a drop in transmission of at least 10% was needed to control infection in the absence of further treatment. For scenario three, even assuming the highest level of efficacy (Figure S19), MDA alone was not sufficient to control infection (Figure S19e&f), without further rounds of MDA and an instantaneous reduction of more than 10%

was needed to control infection. However three rounds of MDA in the mesoendemic community alone was enough to control infection at this high efficacy (Figure S19c&d).

Assuming the lowest treatment efficacy in the hypoendemic community (Figure S20) it was possible to reduce prevalence of active disease to 5% with one annual round MDA for three years (Figure S20b). However after treatment was discontinued prevalence of infection and active disease increased. However with scenario two infection was eliminated within the community following three rounds of treatment (Figure S20c&d). Limited additional reduction in the transmission rate was needed to prevent possible re-emergence of infection in scenario 4 (Figure S20g&h). However > 10% was needed for scenario 3 (Figure S20e&f).

Assuming the highest level of efficacy assessed here infection was completely eliminated from the community with three rounds of MDA under both antibiotic distribution strategies (Figure S21a-d), therefore any additional transmission reduction achieved with scenarios three and four would only be necessary to ensure infection did not re-emerge (Figure S21).

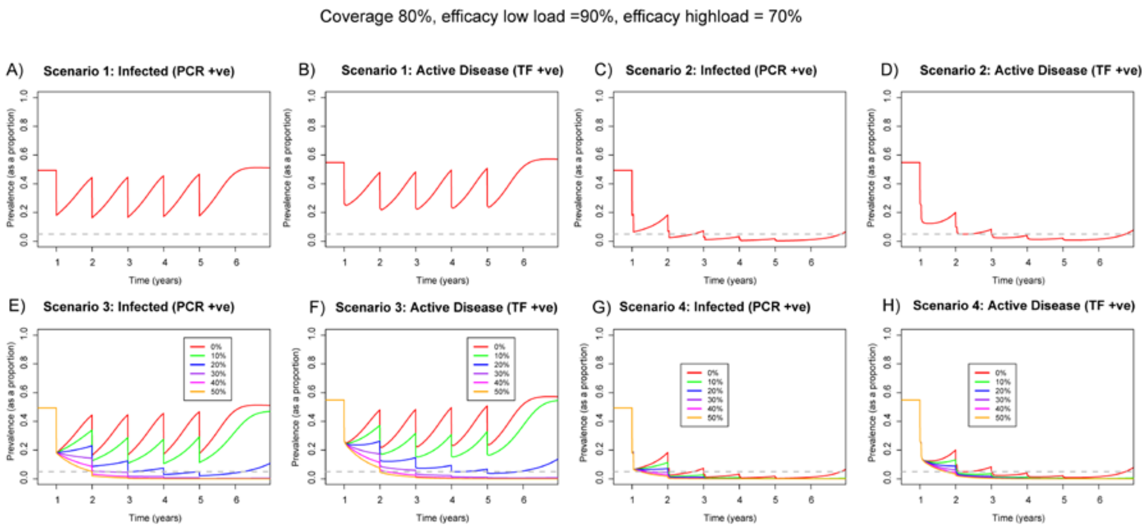

Additional file 1: Figure S16. Prevalence of infection and active disease in 0- 9 year olds when comparing single-dose annual MDA to double-dose annual MDA (conducted two weeks apart) within a hyperendemic community for five years. Assuming 80% coverage and 70% and 90% efficacy of the antibiotic for high and low bacterial loaders respectively. A) prevalence of infection and B) active disease with single-dose annual MDA. C) prevalence of infection and D) active disease with double-dose annual MDA applied two weeks apart. E) prevalence of infection and F) active disease with single-dose annual MDA and G) prevalence of infection and H) active disease with double-dose annual MDA applied two weeks apart, while also modelling an instantaneous drop in β, we present a range of reductions in beta. Different coloured lines represent different percentage declines in the value of β through enhanced F&E, ranging from 0% - 50%. Grey dashed line indicates 5% prevalence, where <5% prevalence of active disease is the GET 2020 target level.

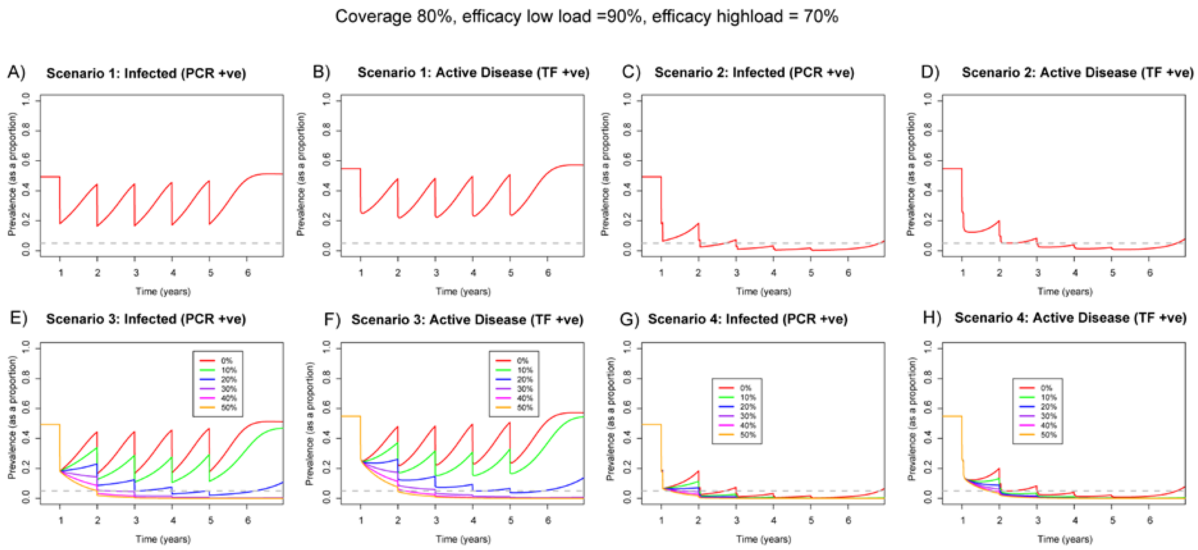

Additional file 1: Figure S17. Prevalence of infection and active disease in 0- 9 year olds when comparing single-dose annual MDA to double-dose annual MDA (conducted two weeks apart) within a hyperendemic community for five years. Assuming 80% coverage and 70% and 90% efficacy of the antibiotic for high and low bacterial loaders respectively. A) prevalence of infection and B) active disease with single-dose annual MDA. C) prevalence of infection and D) active disease with double-dose annual MDA applied two weeks apart. E) prevalence of infection and F) active disease with single-dose annual MDA and G) prevalence of infection and H) active disease with double-dose annual MDA applied two weeks apart, while also modelling an instantaneous drop in β, we present a range of reductions in beta. Different coloured lines represent different percentage declines in the value of β through enhanced F&E, ranging from 0% - 50%. Grey dashed line indicates 5% prevalence, where <5% prevalence of active disease is the GET 2020 target level.

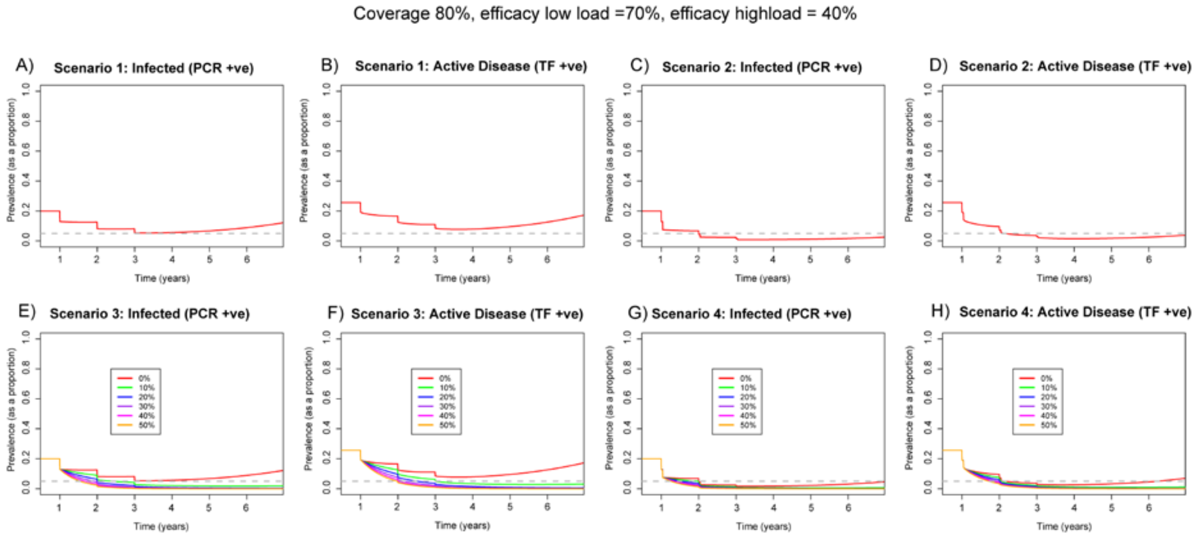

Additional file 1: Figure S18. Prevalence of infection and active disease in 0- 9 year olds when comparing single-dose annual MDA to double-dose annual MDA (conducted two weeks apart) within a mesoendemic community for three years. Assuming 80% coverage and 40% and 70% efficacy of the antibiotic for high and low bacterial loaders respectively. A) prevalence of infection and B) active disease with single-dose annual MDA. C) prevalence of infection and D) active disease with double-dose annual MDA applied two weeks apart.

E) prevalence of infection and F) active disease with single-dose annual MDA and G) prevalence of infection and H) active disease with double-dose annual MDA applied two weeks apart, while also modelling an instantaneous drop in β, we present a range of reductions in beta. Different coloured lines represent different percentage declines in the value of β through enhanced F&E, ranging from 0% - 50%. Grey dashed line indicates 5% prevalence, where <5% prevalence of active disease is the GET 2020 target level.

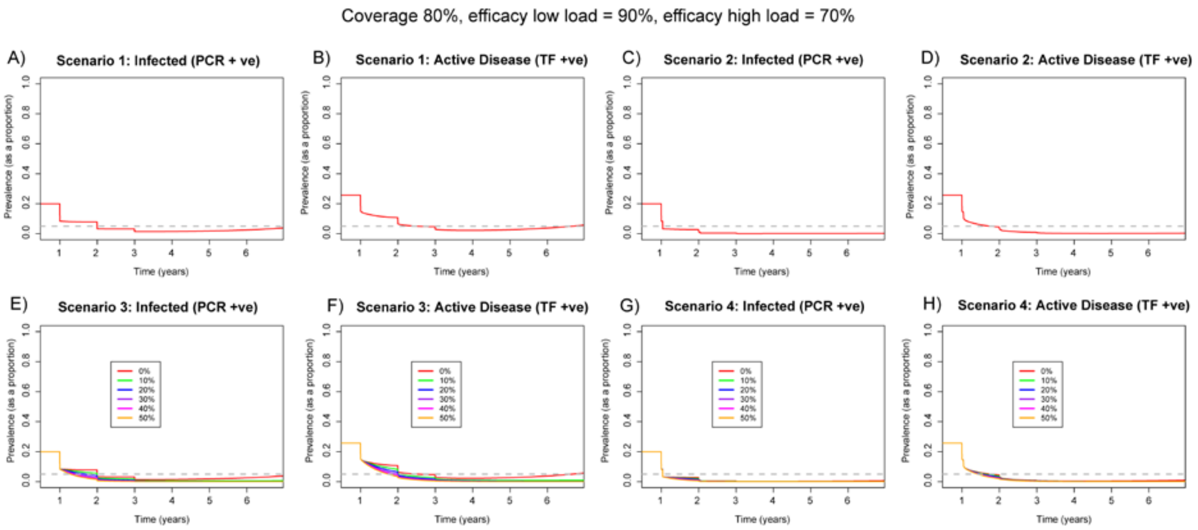

Additional file 1: Figure S19. Prevalence of infection and active disease in 1- 9 year olds when comparing single-dose annual MDA to double-dose annual MDA (conducted two weeks apart) within a mesoendemic community for three years. Assuming 80% coverage and 70% and 90% efficacy of the antibiotic for high and low bacterial loaders respectively. A) prevalence of infection and B) active disease with single-dose annual MDA. C) prevalence of infection and D) active disease with double-dose annual MDA applied two weeks apart. E) prevalence of infection and F) active disease with single-dose annual MDA and G) prevalence of infection and H) active disease with double-dose annual MDA applied two weeks apart, while also modelling an instantaneous drop in β, we present a range of reductions in beta. Different coloured lines represent different percentage declines in the value of β through enhanced F&E, ranging from 0% - 50%. Grey dashed line indicates 5% prevalence, where <5% prevalence of active disease is the GET 2020 target level.

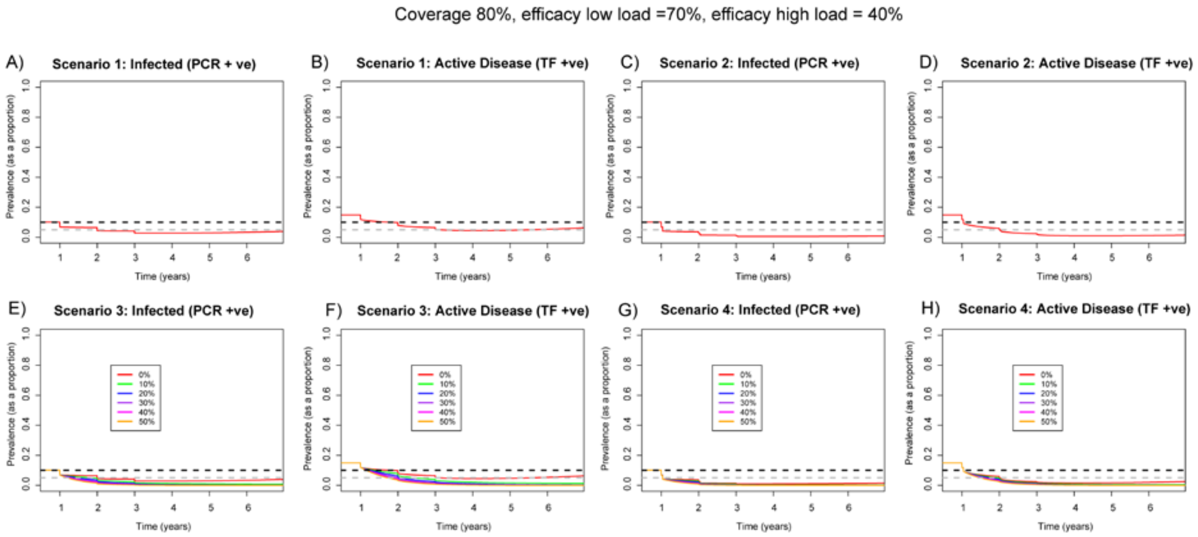

Additional file 1: Figure S20. Prevalence of infection and active disease in 0- 9 year olds when comparing single-dose annual MDA to double-dose annual MDA (conducted two weeks apart) within a hypoendemic community for three years. Assuming 80% coverage and 40% and 70% efficacy of the antibiotic for high and low bacterial loaders respectively. A) prevalence of infection and B) active disease with single-dose annual MDA. C) prevalence of infection and D) active disease with double-dose annual MDA applied two weeks apart. E) prevalence of infection and F) active disease with single-dose annual MDA and G) prevalence of infection and H) active disease with double-dose annual MDA applied two weeks apart, while also modelling an instantaneous drop in β. Different coloured lines represent different percentage declines in the value of β through enhanced F&E, ranging from 0% - 50%. Grey dashed line indicates 5% prevalence, where <5% prevalence of active disease is the GET 2020 target level.

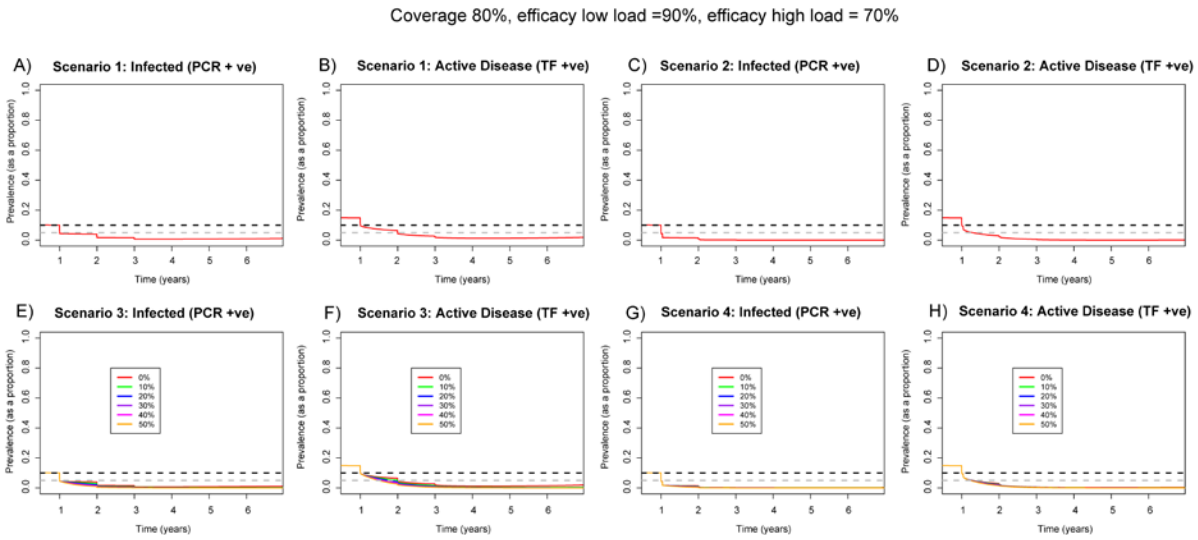

Additional file 1: Figure S21. Prevalence of infection and active disease in 0- 9 year olds when comparing single-dose annual MDA to double-dose annual MDA (conducted two weeks apart) within a hypoendemic community for three years. Assuming 80% coverage and 70% and 90% efficacy of the antibiotic for high and low bacterial loaders respectively. A) prevalence of infection and B) active disease with single-dose annual MDA. C) prevalence of infection and D) active disease with double-dose annual MDA applied two weeks apart. E) prevalence of infection and F) active disease with single-dose annual MDA and G) prevalence of infection and H) active disease with double-dose annual MDA applied two weeks apart, while also modelling an instantaneous drop in β, we present a range of reductions in beta. Different coloured lines represent different percentage declines in the value of β through enhanced F&E, ranging from 0% - 50%. Grey dashed line indicates 5% prevalence, where <5% prevalence of active disease is the GET 2020 target level.

Sensitivity of the results to the number of infections at which immunity plateaus

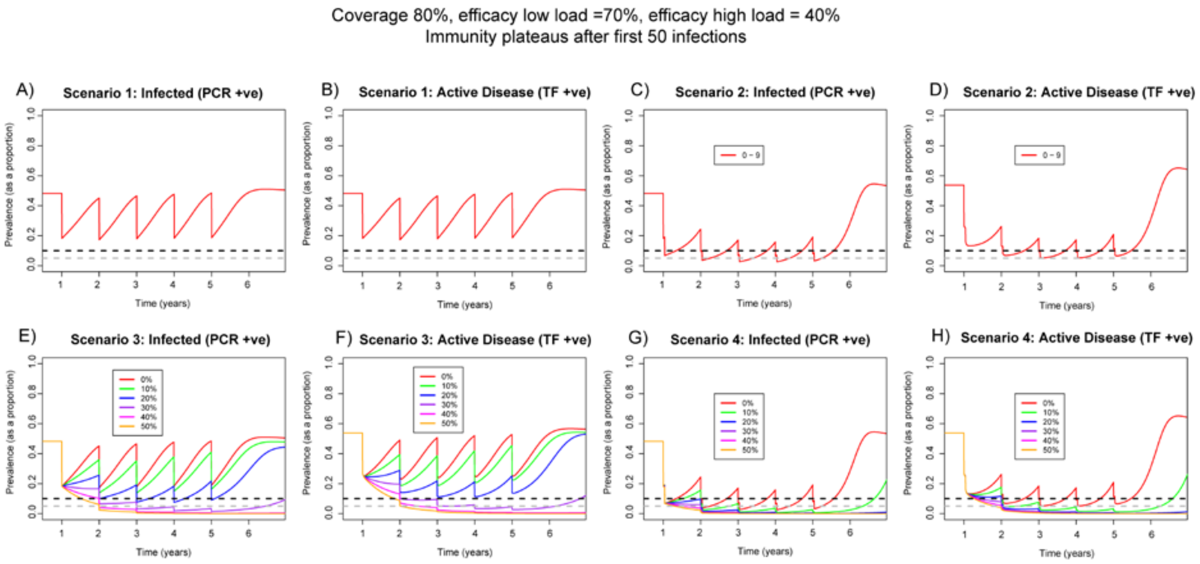

Additional file 1: Figure S22. Prevalence of infection and active disease in 0- 9 year olds when comparing single-dose annual MDA to double-dose annual MDA (conducted two weeks apart) within a hyperendemic community for five years. Assuming immunity plateaus after 50 infections. A) prevalence of infection and B) active disease with single-dose annual MDA. C) prevalence of infection and D) active disease with double-dose annual MDA applied two weeks apart. E) prevalence of infection and F) active disease with single-dose annual MDA and G) prevalence of infection and H) active disease with double-dose annual MDA applied two weeks apart, while also modelling an instantaneous drop in β, we present a range of reductions in beta. Different coloured lines represent different percentage declines in the value of β through enhanced F&E, ranging from 0% - 50%. Grey dashed line indicates 5% prevalence, where <5% prevalence is the GET 2020 target level.

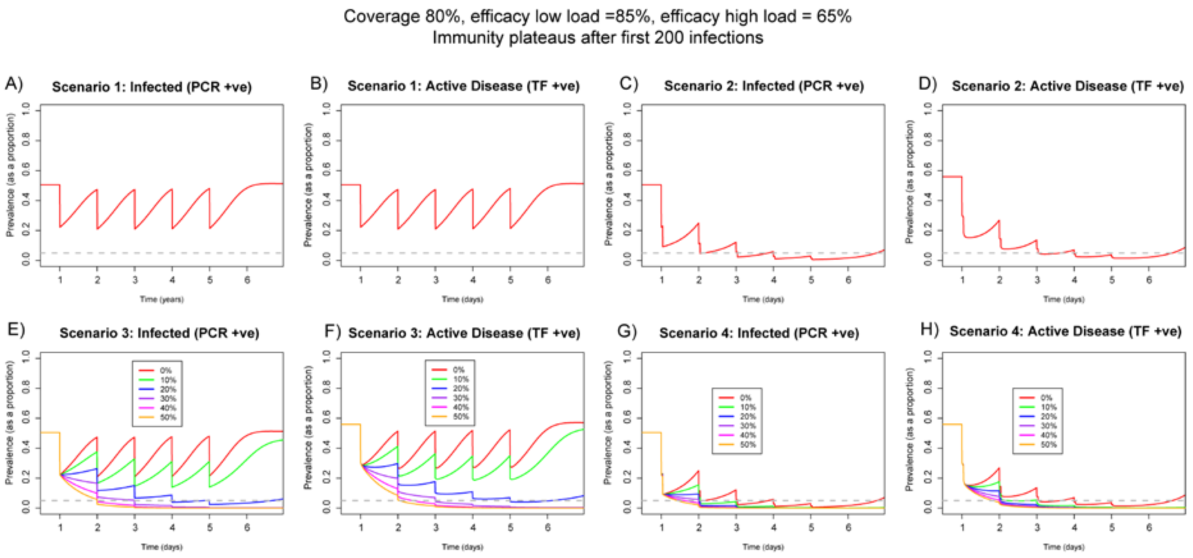

Additional file 1: Figure S23. Prevalence of infection and active disease in 0- 9 year olds when comparing single-dose annual MDA to double-dose annual MDA (conducted two weeks apart) within a hyperendemic community for five years. Assuming immunity plateaus after 200 infections. A) prevalence of infection and B) active disease with single-dose annual MDA. C) prevalence of infection and D) active disease with double-dose annual MDA applied two weeks apart. E) prevalence of infection and F) active disease with single-dose annual MDA and G) prevalence of infection and H) active disease with double-dose annual MDA applied two weeks apart, while also modelling an instantaneous drop in β, we present a range of reductions in beta. Different coloured lines represent different percentage declines in the value of β through enhanced F&E, ranging from 0% - 50%. Grey dashed line indicates 5% prevalence, where <5% prevalence of active disease is the GET 2020 target level.

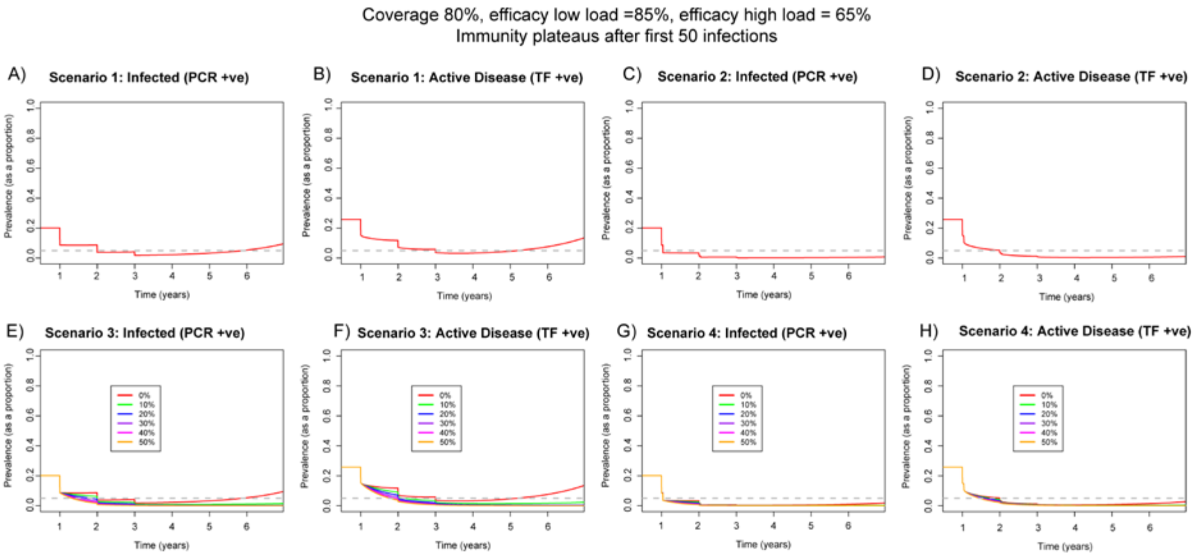

Additional file 1: Figure S24. Prevalence of infection and active disease in 0- 9 year olds when comparing single-dose annual MDA to double-dose annual MDA (conducted two weeks apart) within a mesoendemic community for three years. Assuming immunity plateaus after 50 infections. A) prevalence of infection and B)

active disease with single-dose annual MDA. C) prevalence of infection and D) active disease with double-dose annual MDA applied two weeks apart. E) prevalence of infection and F) active disease with single-dose annual MDA and G) prevalence of infection and H) active disease with double-dose annual MDA applied two weeks apart, while also modelling an instantaneous drop in β, we present a range of reductions in beta. Different coloured lines represent different percentage declines in the value of β through enhanced F&E, ranging from 0% - 50%. Grey dashed line indicates 5% prevalence, where <5% prevalence of active disease is the GET 2020 target level.

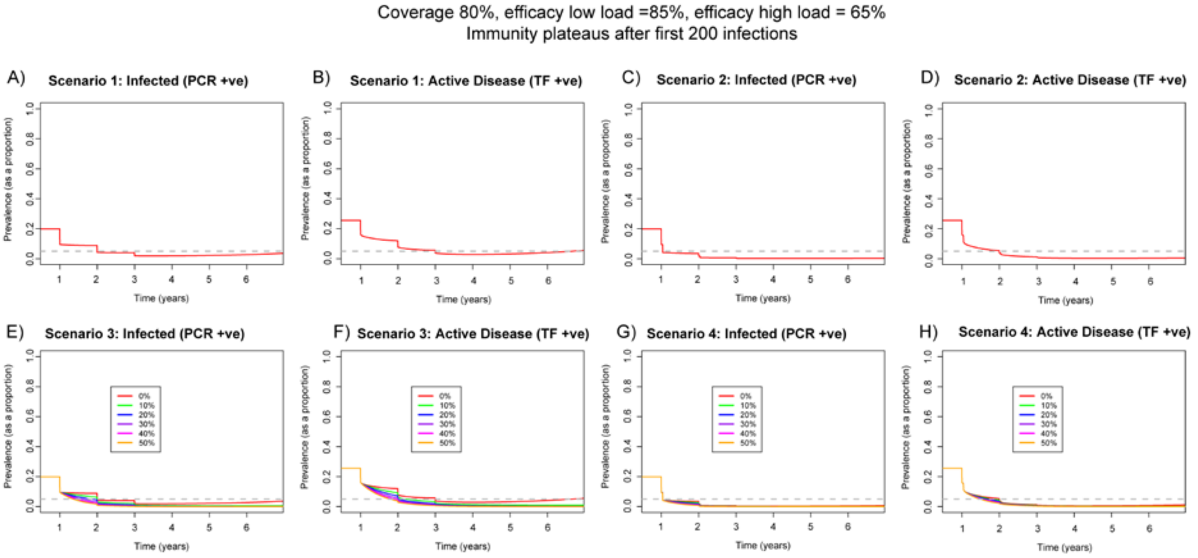

Additional file 1: Figure S25. Prevalence of infection and active disease in 0- 9 year olds when comparing single-dose annual MDA to double-dose annual MDA (conducted two weeks apart) within a mesoendemic community for three years. Assuming immunity plateaus after 200 infections. A) prevalence of infection and B) active disease with single-dose annual MDA. C) prevalence of infection and D) active disease with double-dose annual MDA applied two weeks apart. E) prevalence of infection and F) active disease with single-dose annual MDA and G) prevalence of infection and H) active disease with double-dose annual MDA applied two weeks apart, while also modelling an instantaneous drop in β, we present a range of reductions in beta. Different coloured lines represent different percentage declines in the value of β through enhanced F&E, ranging from 0% - 50%. Grey dashed line indicates 5% prevalence, where <5% prevalence of active disease is the GET 2020 target level.

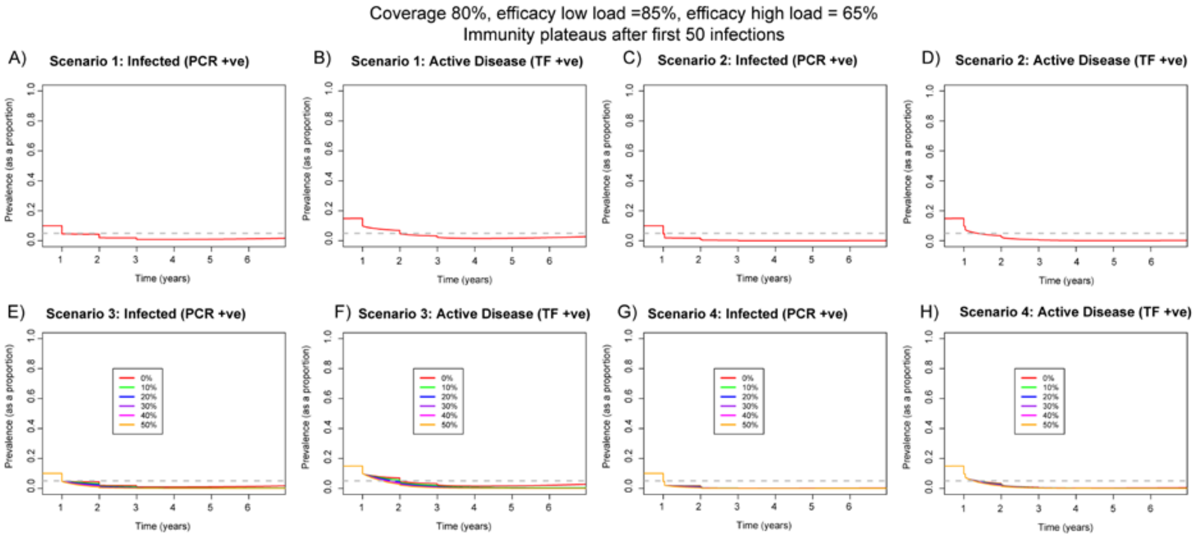

Additional file 1: Figure S26. Prevalence of infection and active disease in 0- 9 year olds when comparing single-dose annual MDA to double-dose annual MDA (conducted two weeks apart) within a hypoendemic community for three years. Assuming immunity plateaus after 50 infections. A) prevalence of infection and B) active disease with single-dose annual MDA. C) prevalence of infection and D) active disease with double-dose annual MDA applied two weeks apart. E) prevalence of infection and F) active disease with single-dose annual MDA and G) prevalence of infection and H) active disease with double-dose annual MDA applied two weeks apart, while also modelling an instantaneous drop in β, we present a range of reductions in beta. Different coloured lines represent different percentage declines in the value of β through enhanced F&E, ranging from 0% - 50%. Grey dashed line indicates 5% prevalence, where <5% prevalence of active disease is the GET 2020 target level.

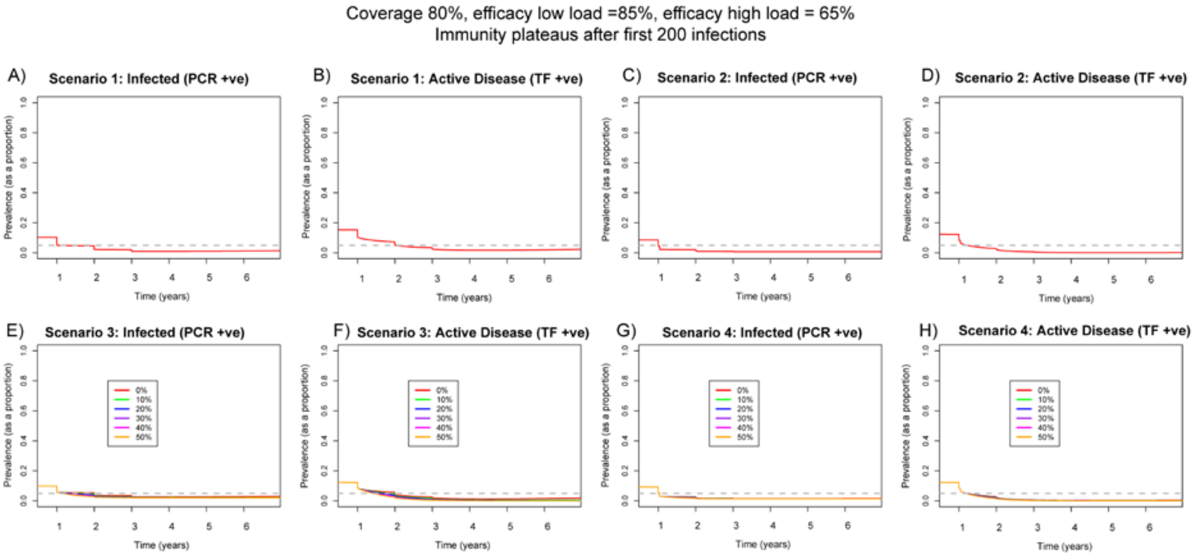

Additional file 1: Figure S27. Prevalence of infection and active disease in 0- 9 year olds when comparing single-dose annual MDA to double-dose annual MDA (conducted two weeks apart) within a hypoendemic community for three years. Assuming immunity plateaus after 200 infections. A) prevalence of infection and B) active disease with single-dose annual MDA. C) prevalence of infection and D) active disease with double-dose

annual MDA applied two weeks apart. E) prevalence of infection and F) active disease with single-dose annual MDA and G) prevalence of infection and H) active disease with double-dose annual MDA applied two weeks apart, while also modelling an instantaneous drop in β, we present a range of reductions in beta. Different coloured lines represent different percentage declines in the value of β through enhanced F&E, ranging from 0% - 50%. Grey dashed line indicates 5% prevalence, where <5% prevalence of active disease is the GET 2020 target level.

Sensitivity of the results to variation in the number of infections required for immunity to plateau

Increasing the number of infections at which immunity plateaus did not result in any dramatic differences between the scenarios modelled. However, it was apparent that reducing the number of infections at which immunity plateaued resulted in much more rapid re-emergence following each round of infection within the community, while increasing the number resulted in a much slower re-emergence of infection. This trend was most apparent for the hyperendemic community, while only a very small difference was observed for the hypoendemic community (Figure S26-S27).

<u>Sensitivity of the results to changes in the number of high bacterial loaders</u>

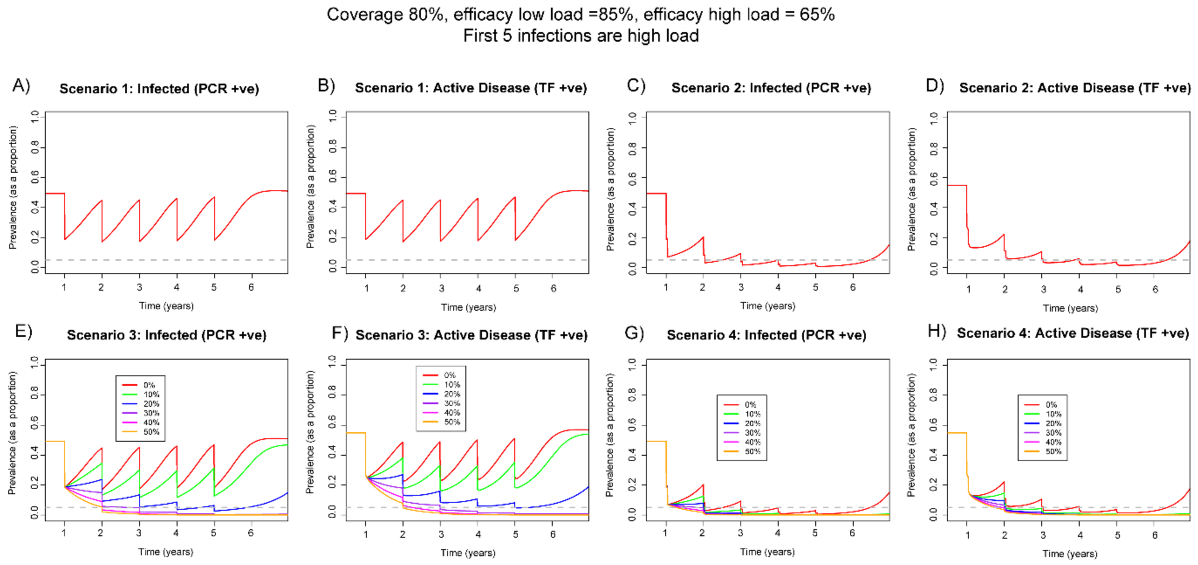

Additional file 1: Figure S28. Prevalence of infection and active disease in 0- 9 year olds when comparing single-dose annual MDA to double-dose annual MDA (conducted two weeks apart) within a hyperendemic community for five years. Assuming the first 5 infections are high load infections. A) prevalence of infection and B) active disease with single-dose annual MDA. C) prevalence of infection and D) active disease with double-dose annual MDA applied two weeks apart. E) prevalence of infection and F) active disease with single-dose annual MDA and G) prevalence of infection and H) active disease with double-dose annual MDA applied two weeks apart, while also modelling an instantaneous drop in β, we present a range of reductions in beta. Different coloured lines represent different percentage declines in the value of β through enhanced F&E, ranging from 0% - 50%. Grey dashed line indicates 5% prevalence, where <5% prevalence of active disease is the GET 2020 target level.

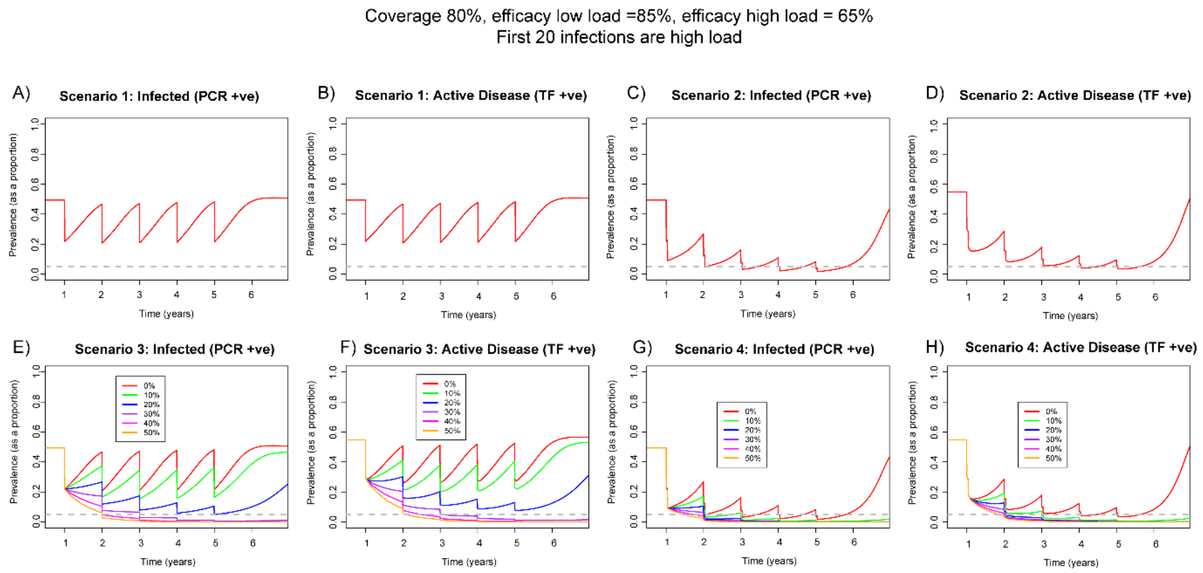

Additional file 1: Figure S29. Prevalence of infection and active disease in 0- 9 year olds when comparing single-dose annual MDA to double-dose annual MDA (conducted two weeks apart) within a hyperendemic community for five years. Assuming the first 20 infections are high load infections. A) prevalence of infection and B) active disease with single-dose annual MDA. C) prevalence of infection and D) active disease with double-dose annual MDA applied two weeks apart. E) prevalence of infection and F) active disease with single-dose annual MDA and G) prevalence of infection and H) active disease with double-dose annual MDA applied two weeks apart, while also modelling an instantaneous drop in β, we present a range of reductions in beta. Different coloured lines represent different percentage declines in the value of β through enhanced F&E, ranging from 0% - 50%. Grey dashed line indicates 5% prevalence, where <5% prevalence of active disease is the GET 2020 target level.

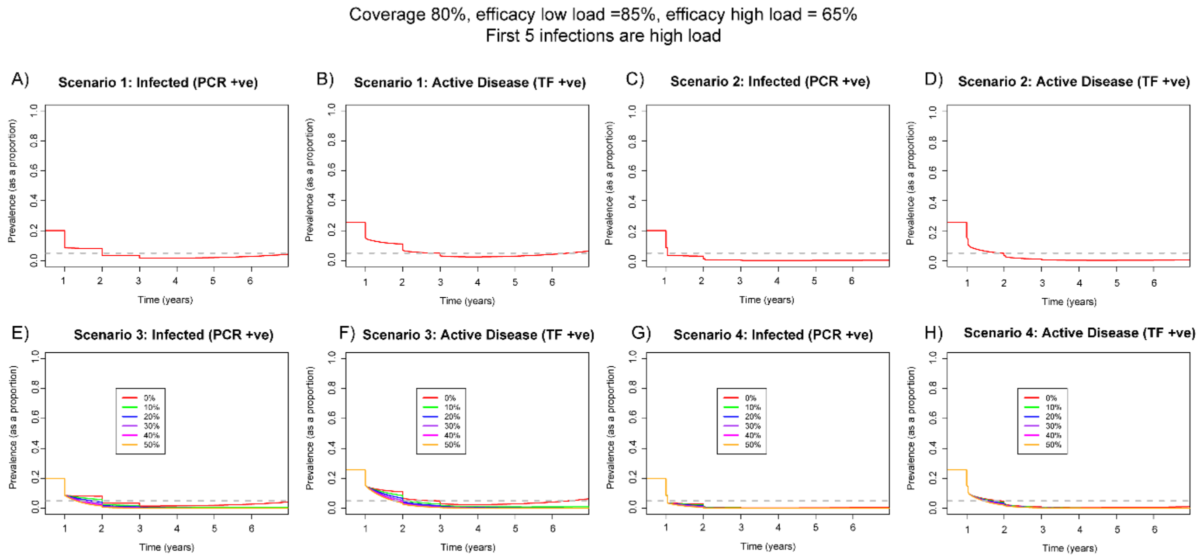

Additional file 1: Figure S30. Prevalence of infection and active disease in 0- 9 year olds when comparing single-dose annual MDA to double-dose annual MDA (conducted two weeks apart) within a mesoendemic community for three years. Assuming the first 5 infections are high load infections. A) prevalence of infection

and B) active disease with single-dose annual MDA. C) prevalence of infection and D) active disease with double-dose annual MDA applied two weeks apart. E) prevalence of infection and F) active disease with single-dose annual MDA and G) prevalence of infection and H) active disease with double-dose annual MDA applied two weeks apart, while also modelling an instantaneous drop in β, we present a range of reductions in beta. Different coloured lines represent different percentage declines in the value of β through enhanced F&E, ranging from 0% - 50%. Grey dashed line indicates 5% prevalence, where <5% prevalence of active disease is the GET 2020 target level.

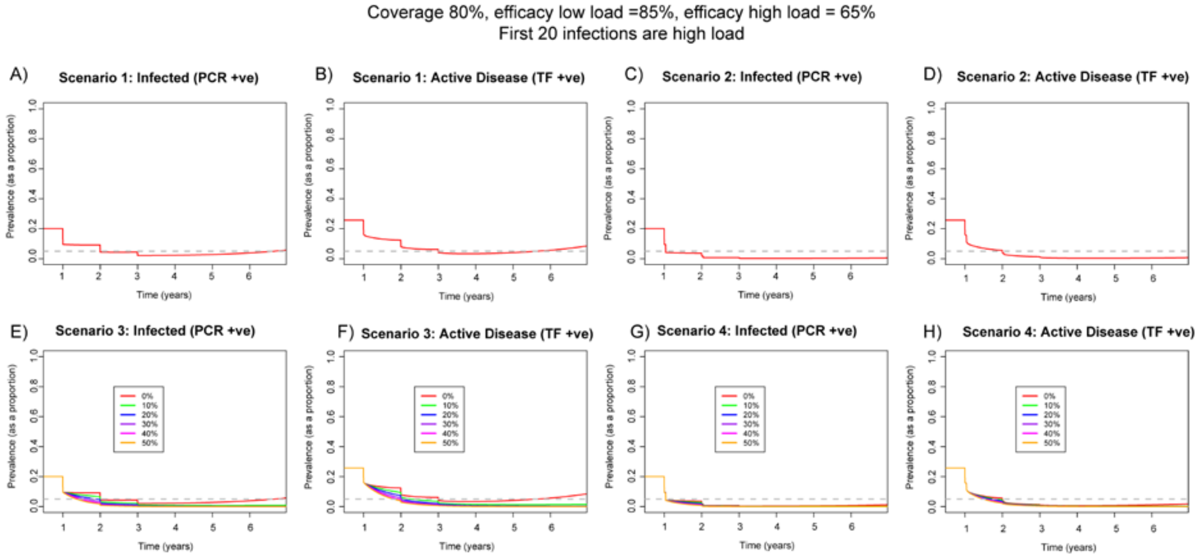

Additional file 1: Figure S31. Prevalence of infection and active disease in 0- 9 year olds when comparing single-dose annual MDA to double-dose annual MDA (conducted two weeks apart) within a mesoendemic community for three years. Assuming the first 20 infections are high load infections. A) prevalence of infection and B) active disease with single-dose annual MDA. C) prevalence of infection and D) active disease with double-dose annual MDA applied two weeks apart. E) prevalence of infection and F) active disease with single-dose annual MDA and G) prevalence of infection and H) active disease with double-dose annual MDA applied two weeks apart, while also modelling an instantaneous drop in β, we present a range of reductions in beta. Different coloured lines represent different percentage declines in the value of β through enhanced F&E, ranging from 0% - 50%. Grey dashed line indicates 5% prevalence, where <5% prevalence of active disease is the GET 2020 target level.

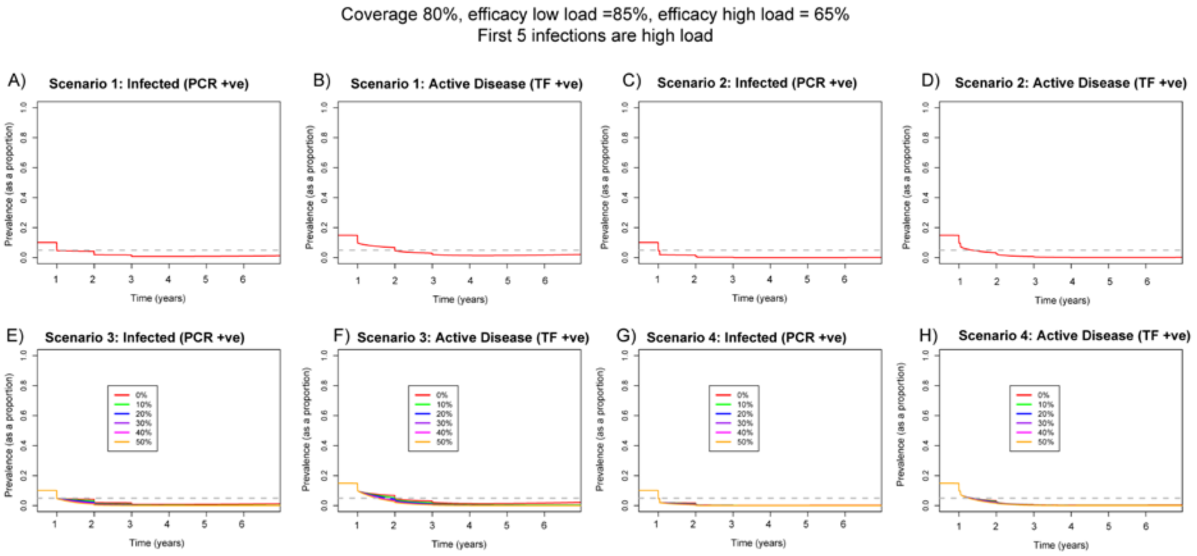

Additional file 1: Figure S32. Prevalence of infection and active disease in 0- 9 year olds when comparing single-dose annual MDA to double-dose annual MDA (conducted two weeks apart) within a hypoendemic community for three years. Assuming the first 5 infections are high load infections. A) prevalence of infection and B) active disease with single-dose annual MDA. C) prevalence of infection and D) active disease with double-dose annual MDA applied two weeks apart. E) prevalence of infection and F) active disease with single-dose annual MDA and G) prevalence of infection and H) active disease with double-dose annual MDA applied two weeks apart, while also modelling an instantaneous drop in β, we present a range of reductions in beta. Different coloured lines represent different percentage declines in the value of β through enhanced F&E, ranging from 0% - 50%. Grey dashed line indicates 5% prevalence, where <5% prevalence of active disease is the GET 2020 target level.

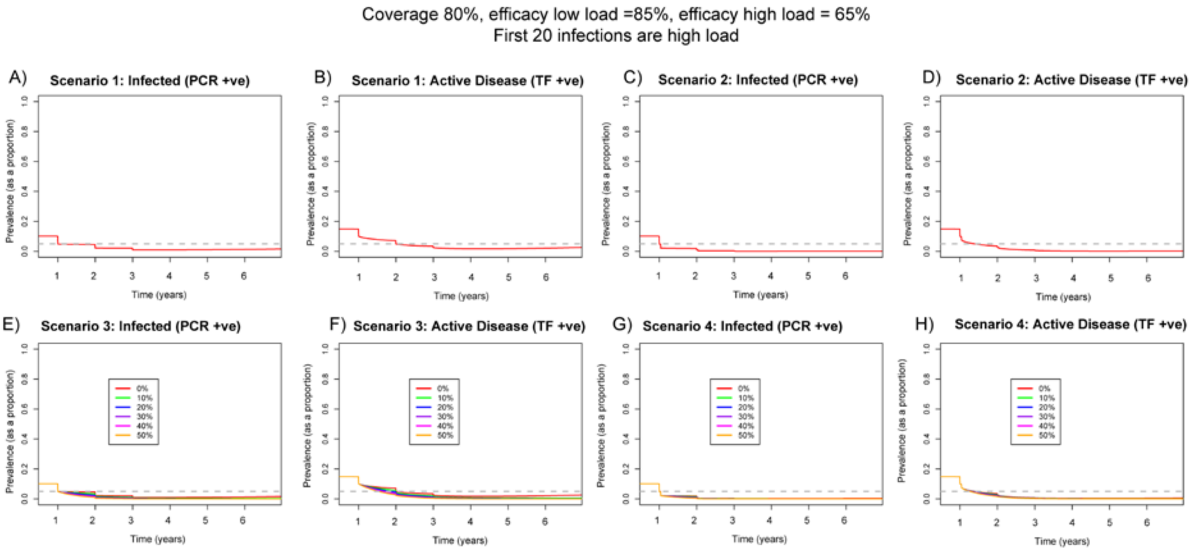

Additional file 1: Figure S33. Prevalence of infection and active disease in 0- 9 year olds when comparing single-dose annual MDA to double-dose annual MDA (conducted two weeks apart) within a hypoendemic community for three years. Assuming the first 20 infections are high load infections. A) prevalence of infection and B) active disease with single-dose annual MDA. C) prevalence of infection and D) active disease with

double-dose annual MDA applied two weeks apart. E) prevalence of infection and F) active disease with single-dose annual MDA and G) prevalence of infection and H) active disease with double-dose annual MDA applied two weeks apart, while also modelling an instantaneous drop in β, we present a range of reductions in beta. Different coloured lines represent different percentage declines in the value of β through enhanced F&E, ranging from 0% - 50%. Grey dashed line indicates 5% prevalence, where <5% prevalence of active disease is the GET 2020 target level.

Sensitivity of the results to variation in the number of high load infections

For all transmission settings limited impact on the effectiveness of the interventions was observed when we varied the number of infections that qualified individuals as either high or low loaders. We saw a small change in the dynamics of infection treatment and re-emergence for the hyperendemic community only (Figures S28 – S33). When the first 20 infections were assumed to be high loaders we saw a slightly larger impact on the reduction in prevalence with the double-dose treatment. However, we also observed a faster rate of re-emergence following treatment cessation when the first 20 infections were classified as high load. Very similar levels of transmission reduction through enhanced F&E were required for the long-term control of infection. This effect was most apparent for the hyperendemic community (Figure S28&S29), and least apparent for the hypoendemic community (Figure S32&S33).

Sensitivity of the results to a non-instantaneous large reduction in β

It is possible that F&E will not be accessed by all members of the community equally, and at the same rate. Therefore we also consider incremental reductions in β occur when each round of MDA does. We assume that the decline in β is exponential, therefore the largest reduction in β occurs when the first round of MDA does, but that additional reductions occur in β across the trial period. The same total reduction in β is modelled, however the time taken to achieve this total reduction is longer in the findings presented below.

Overall, we identified very little difference in the results between the two different reductions in β for the meso (Figure S35) and hypoendemic communities (Figure S35). However, for the hyperendemic community (Figure S34) the year-on-year reductions in infection prevalence seen with the double dose strategy and with annual MDA and enhanced F&E were smaller than when an instantaneous reduction was assumed. However, the overall findings remained consistent.

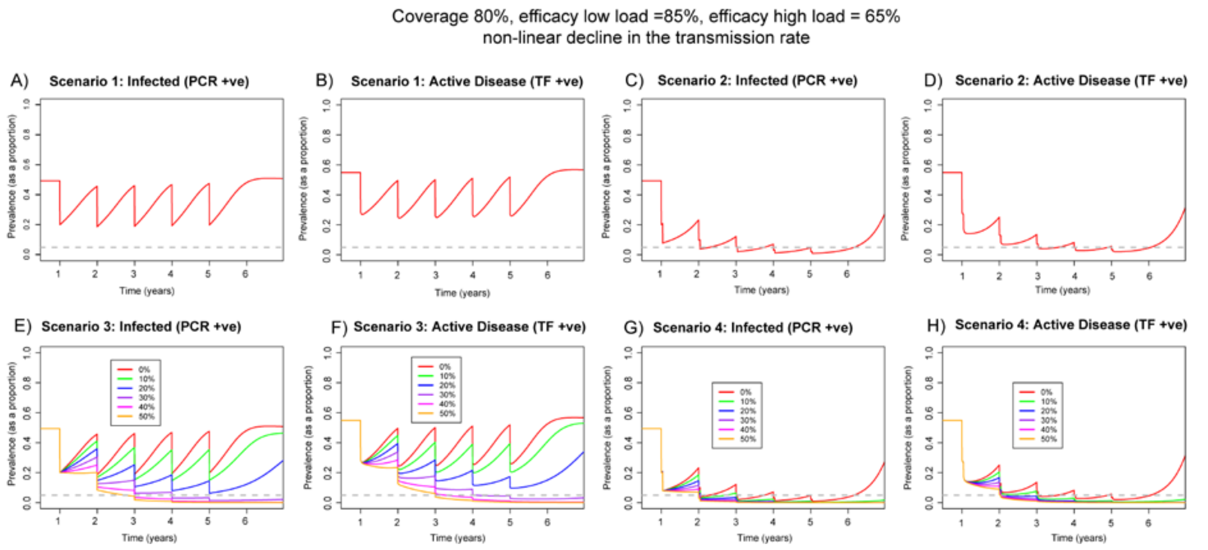

Additional file 1: Figure S34. Prevalence of infection and active disease in 0- 9 year olds when comparing single-dose annual MDA to double-dose annual MDA (conducted two weeks apart) within a hyperendemic community for five years. Assuming a non-instantaneous decline in β through enhanced F&E. A) prevalence of infection and B) active disease with single-dose annual MDA. C) prevalence of infection and D) active disease with double-dose annual MDA applied two weeks apart. E) prevalence of infection and F) active disease with single-dose annual MDA and G) prevalence of infection and H) active disease with double-dose annual MDA applied two weeks apart, while also modelling an instantaneous drop in β, we present a range of reductions in beta. Different coloured lines represent different percentage declines in the value of β through enhanced F&E that occur across the 5 year intervention period, ranging from 0% - 50%. Grey dashed line indicates 5% prevalence, where <5% prevalence of active disease is the GET 2020 target level.

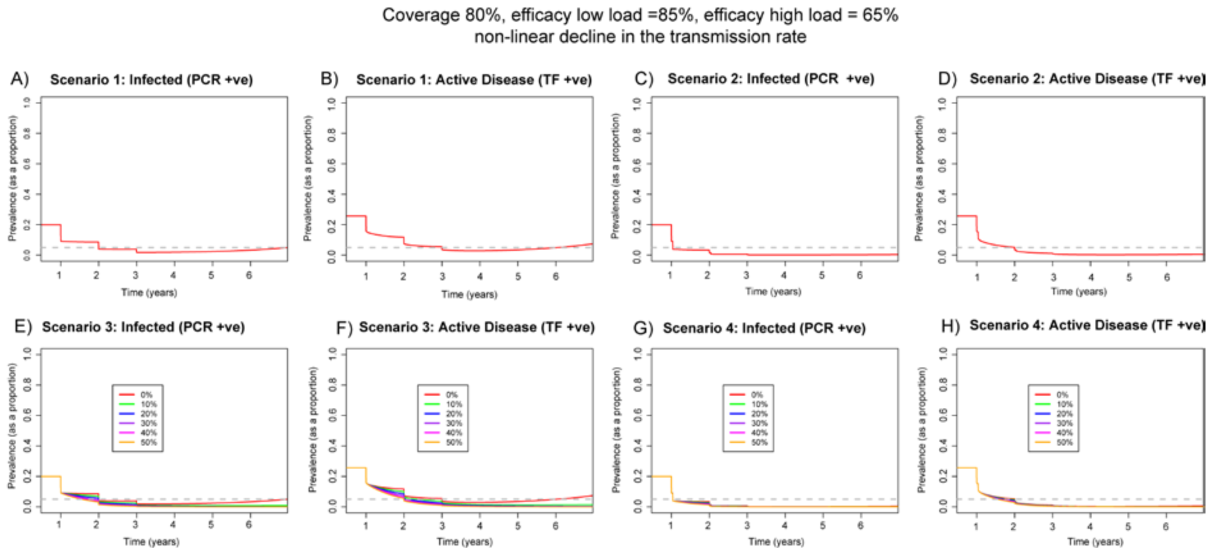

Additional file 1: Figure S35. Prevalence of infection and active disease in 0- 9 year olds when comparing single-dose annual MDA to double-dose annual MDA (conducted two weeks apart) within a mesoendemic community for three years. Assuming a non-instantaneous decline in β through enhanced F&E. A) prevalence of infection and B) active disease with single-dose annual MDA. C) prevalence of infection and D) active

disease with double-dose annual MDA applied two weeks apart. E) prevalence of infection and F) active disease with single-dose annual MDA and G) prevalence of infection and H) active disease with double-dose annual MDA applied two weeks apart, while also modelling an instantaneous drop in β, we present a range of reductions in beta. Different coloured lines represent different percentage declines in the value of β through enhanced F&E that occur across the 5 year intervention period, ranging from 0% - 50%. Grey dashed line indicates 5% prevalence, where <5% prevalence of active disease is the GET 2020 target level.

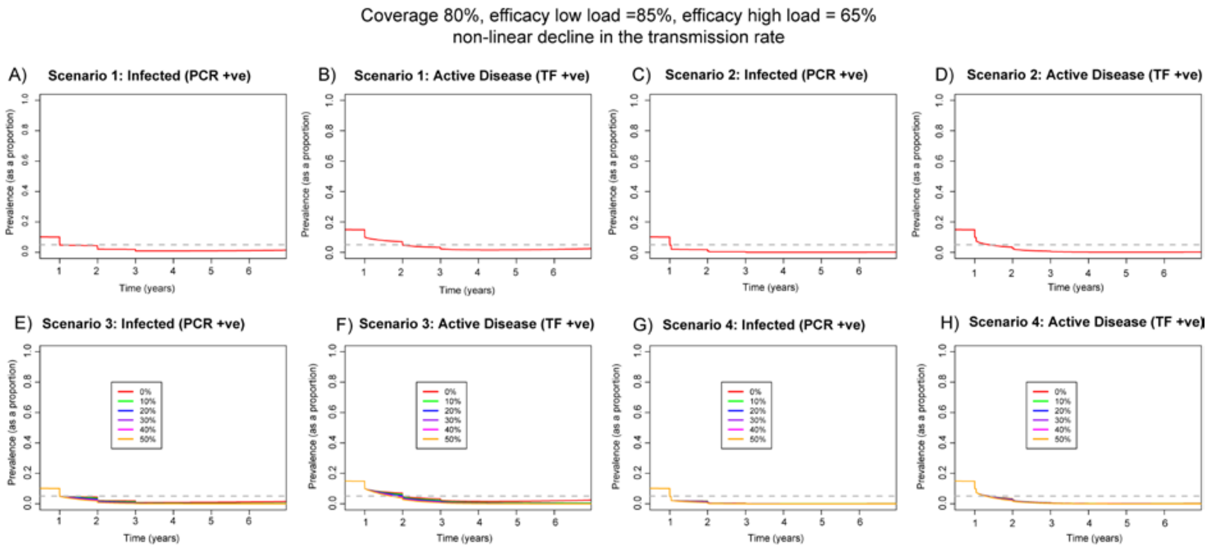

Additional file 1: Figure S36. Prevalence of infection and active disease in 0- 9 year olds when comparing single-dose annual MDA to double-dose annual MDA (conducted two weeks apart) within a hypoendemic community for three years. Assuming a non-instantaneous decline in β through enhanced F&E. A) prevalence of infection and B) active disease with single-dose annual MDA. C) prevalence of infection and D) active disease with double-dose annual MDA applied two weeks apart. E) prevalence of infection and F) active disease with single-dose annual MDA and G) prevalence of infection and H) active disease with double-dose annual MDA applied two weeks apart, while also modelling an instantaneous drop in β, we present a range of reductions in beta. Different coloured lines represent different percentage declines in the value of β through enhanced F&E that occur across the 5 year intervention period, ranging from 0% - 50%. Grey dashed line indicates 5% prevalence, where <5% prevalence of active disease is the GET 2020 target level.

Sensitivity of the results to an increase in the susceptibility to re-infection in the AD state

In the baseline model we assumed that individuals in the AD state (TF positive only) were 100% immune to re-infection. We test the sensitivity of our results to this assumption and allow individuals to be re-infected in the AD state with a 50% probability.

Increasing the susceptibility of individuals in the AD state to re-infection results in the value of beta required to achieve a given level of endemic prevalence is reduced for all transmission settings. Thus, in all transmission settings the effort in terms of interventions required to eliminate infection within the community is less than the baseline analysis. For example, in the hyperendemic community (Figure S37 c-d), treatment with the

double-dose strategy alone was sufficient to eliminate infection from the community, without any F&E. While with annual MDA alone, yearly gains in prevalence reduction of infection were also observed in the hyperendemic community (Figure S37 a-b) – however this was still insufficient to eliminate infection in the absence of additional rounds of MDA. While for the mesoendemic (Figure S38) and hypoendemic (Figure S39) settings successful elimination was achieved under all scenarios, with limited to no evidence of infection re-emergence at the end of the follow-up period.

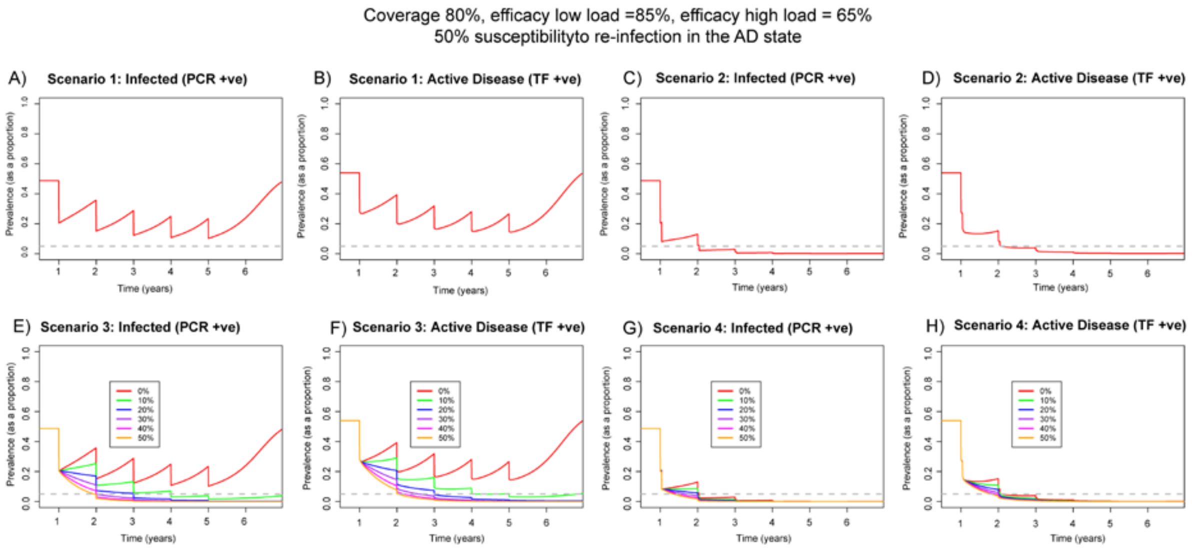

Additional file 1: Figure S37. Prevalence of infection and active disease in 0- 9 year olds when comparing single-dose annual MDA to double-dose annual MDA (conducted two weeks apart) within a hyperendemic community for five years. Assuming 50% susceptibility to re-infection in the AD state . A) prevalence of infection and B) active disease with single-dose annual MDA. C) prevalence of infection and D) active disease with double-dose annual MDA applied two weeks apart. E) prevalence of infection and F) active disease with single-dose annual MDA and G) prevalence of infection and H) active disease with double-dose annual MDA applied two weeks apart, while also modelling an instantaneous drop in β, we present a range of reductions in beta. Different coloured lines represent different percentage declines in the value of β through enhanced F&E that occur across the 5 year intervention period, ranging from 0% - 50%. Grey dashed line indicates 5% prevalence, where <5% prevalence of active disease is the GET 2020 target level.

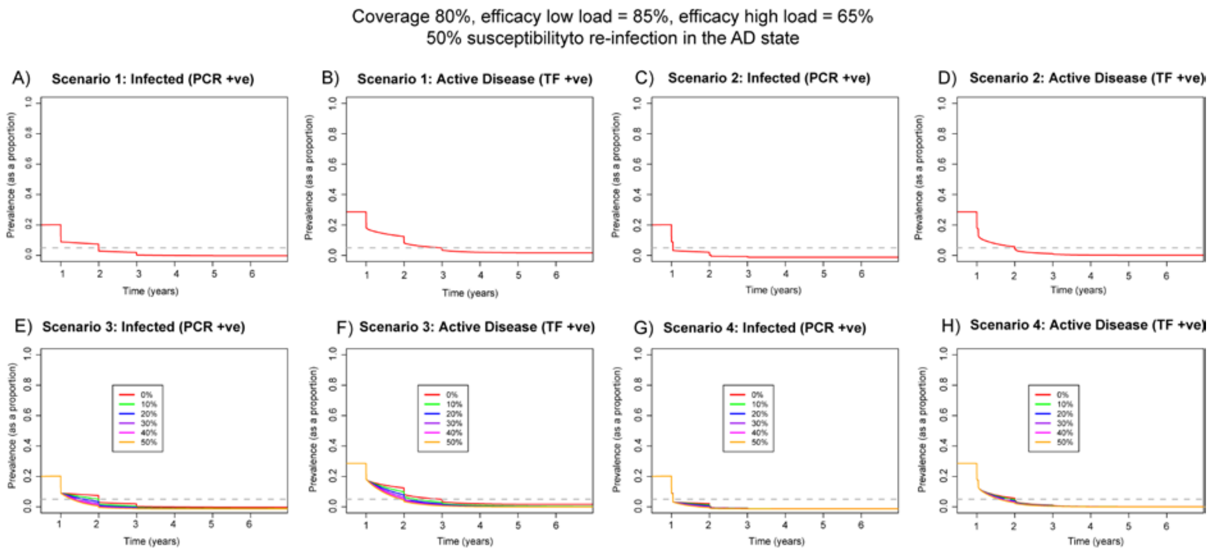

Additional file 1: Figure S38. Prevalence of infection and active disease in 0- 9 year olds when comparing single-dose annual MDA to double-dose annual MDA (conducted two weeks apart) within a mesoendemic community for three years. Assuming 50% susceptibility to re-infection in the AD state . A) prevalence of infection and B) active disease with single-dose annual MDA. C) prevalence of infection and D) active disease with double-dose annual MDA applied two weeks apart. E) prevalence of infection and F) active disease with single-dose annual MDA and G) prevalence of infection and H) active disease with double-dose annual MDA applied two weeks apart, while also modelling an instantaneous drop in β, we present a range of reductions in beta. Different coloured lines represent different percentage declines in the value of β through enhanced F&E that occur across the 5 year intervention period, ranging from 0% - 50%. Grey dashed line indicates 5% prevalence, where <5% prevalence of active disease is the GET 2020 target level.

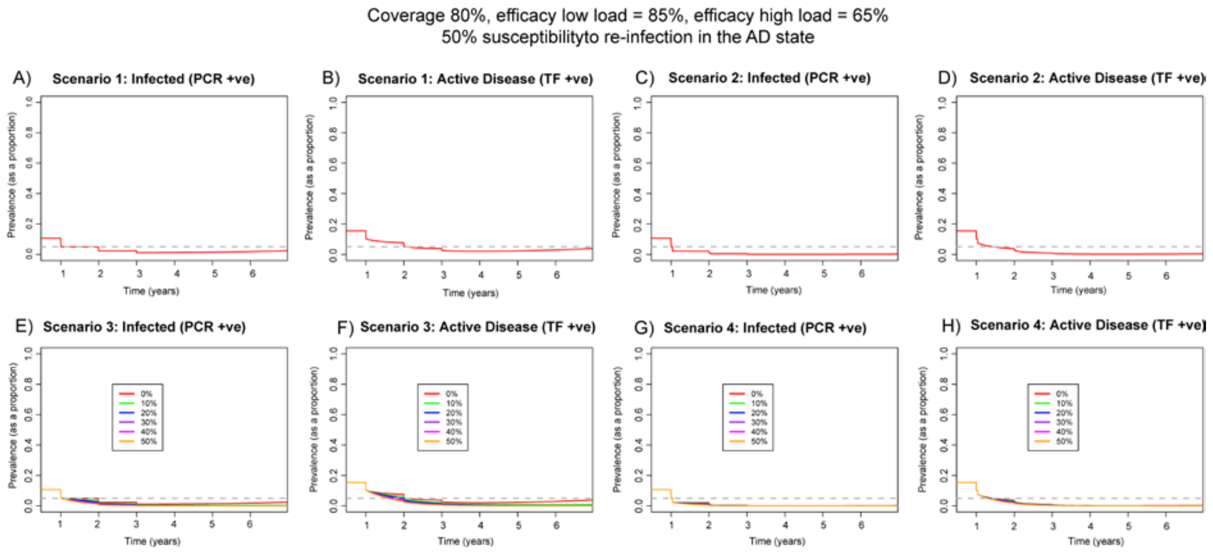

Additional file 1: Figure S39. Prevalence of infection and active disease in 0- 9 year olds when comparing single-dose annual MDA to double-dose annual MDA (conducted two weeks apart) within a hypoendemic community for three years. Assuming 50% susceptibility to re-infection in the AD state . A) prevalence of infection and B) active disease with single-dose annual MDA. C) prevalence of infection and D) active disease

with double-dose annual MDA applied two weeks apart. E) prevalence of infection and F) active disease with single-dose annual MDA and G) prevalence of infection and H) active disease with double-dose annual MDA applied two weeks apart, while also modelling an instantaneous drop in β, we present a range of reductions in beta. Different coloured lines represent different percentage declines in the value of β through enhanced F&E that occur across the 5 year intervention period, ranging from 0% - 50%. Grey dashed line indicates 5% prevalence, where <5% prevalence of active disease is the GET 2020 target level.

References

1. Gambhir M, Basanez MG, Turner F, Kumaresan J, Grassly NC: **Trachoma: transmission, infection, and control**. *Lancet Infec Dis* 2007, **7**:420-427.
2. Gambhir M, Basanez MG, Burton MJ, Solomon AW, Bailey RL, Holland MJ, Blake IM, Donnelly CA, Jabr I, Mabey DC *et al*: **The development of an age-structured model for trachoma transmission dynamics, pathogenesis and control**. *PLoS Negl Trop Dis* 2009, **3**:e462.
3. Lietman TM, Gebre T, Ayele B, Ray KJ, Maher MC, See CW, Emerson PM, Porco TC: **The epidemiological dynamics of infectious trachoma may facilitate elimination**. *Epidemics* 2011, **3**(2):119-124.
4. Anderson R, May R: **Infectious diseases of humans: dynamics and control**: Oxford University Press.
5. Solomon AW, Holland MJ, Burton MJ, West SK, Alexander NDE, Aguirre A, Massae PA, Mkocha H, Muñoz B, Johnson GJ *et al*: **Strategies for control of trachoma: observational study with quantitative PCR**. *Lancet* 2003, **362**:198-204.
6. West ES, Munoz B, Mkocha H, Holland MJ, Aguirre A, Solomon AW, Bailey R, Foster A, Mabey D, West SK: **Mass treatment and the effect on the load of Chlamydia trachomatis infection in a trachoma-hyperendemic community**. *Invest Ophthal & Vis Sci* 2005, **46**:83-87.
7. Faal N, Bailey RL, Jeffries D, Joof H, Sarr I, Laye M, Mabey DCW, Holland MJ: **Conjunctival *FOXP3* Expression in Trachoma: Do Regulatory T Cells Have a Role in Human Ocular *Chlamydia trachomatis* Infection?** *PLoS Med* 2006, **3**:e266.
8. West SK, Munoz B, Mkocha H, Holland MJ, Aguirre A, Solomon AW, Foster A, Bailey RL, Mabey DC: **Infection with Chlamydia trachomatis after mass treatment of a trachoma hyperendemic community in Tanzania: a longitudinal study**. *Lancet* 2005, **366**:1296-1300.
9. Liu F, Porco TC, Mkocha HA, Munoz B, Ray KJ, Bailey RL, Lietman TM, West SK: **The efficacy of oral azithromycin in clearing ocular chlamydia: mathematical modeling from a community-randomized trachoma trial**. *Epidemics* 2014, **6**:10-17.
10. Keenan JD, Ayele B, Gebre T, Zerihun M, Zhou Z, House JI, Gaynor BD, Porco TC, Emerson PM, Lietman TM: **Childhood Mortality in a Cohort Treated With Mass Azithromycin for Trachoma**. *Clin Infect Dis* 2011, **52**:883-888.
11. Grassly NC, Ward ME, Ferris S, Mabey DC, Bailey RL: **The Natural History of Trachoma Infection and Disease in a Gambian Cohort with Frequent Follow-Up**. *PLoS Negl Trop Dis* 2008, **2**:e341.
